# Locating Medical Information during an Infodemic: Information Seeking Behavior and Strategies of Health-Care Workers in Germany

**DOI:** 10.3390/healthcare11111602

**Published:** 2023-05-30

**Authors:** Christopher Holzmann-Littig, David Stadler, Maria Popp, Peter Kranke, Falk Fichtner, Christoph Schmaderer, Lutz Renders, Matthias Christoph Braunisch, Tarek Assali, Louise Platen, Marjo Wijnen-Meijer, Julia Lühnen, Anke Steckelberg, Lisa Pfadenhauer, Bernhard Haller, Cornelia Fuetterer, Christian Seeber, Christian Schaaf, on behalf of the CEOsys Consortium

**Affiliations:** 1 Department of Nephrology, School of Medicine, Klinikum rechts der Isar, Technical University of Munich, 81675 Munich, Germany; david.stadler@mri.tum.de (D.S.); christoph.schmaderer@mri.tum.de (C.S.); lutz.renders@mri.tum.de (L.R.); matthias.braunisch@mri.tum.de (M.C.B.); trassali91@gmail.com (T.A.); louise.platen@mri.tum.de (L.P.); christian.schaaf@mri.tum.de (C.S.); 2 TUM Medical Education Center, School of Medicine, Technical University of Munich, 81675 Munich, Germany; marjo.wijnen-meijer@tum.de; 3 Department of Anaesthesiology, Intensive Care, Emergency and Pain Medicine, University Hospital Würzburg, 97080 Wuerzburg, Germany; popp_m4@ukw.de (M.P.); kranke_p@ukw.de (P.K.); 4 Faculty of Medicine, Clinic and Polyclinic for Anesthesiology and Intensive Care, University of Leipzig, 04103 Leipzig, Germany; falk.fichtner@medizin.uni-leipzig.de (F.F.); christian.seeber@medizin.uni-leipzig.de (C.S.); 5 Institute for Health and Nursing Science, Medical Faculty, Martin Luther University Halle-Wittenberg, 06112 Halle (Saale), Germany; julia.luehnen@uk-halle.de (J.L.); anke.steckelberg@uk-halle.de (A.S.); 6 Clinic for Internal Medicine I, Martin Luther University Halle-Wittenberg, 06112 Halle (Saale), Germany; 7 Institute for Medical Information Processing, Biometry and Epidemiology—IBE, Chair of Public Health and Health Services Research, LMU Munich, 81377 Munich, Germany; pfadenhauer@ibe.med.uni-muenchen.de; 8 Pettenkofer School of Public Health, 81377 Munich, Germany; 9 Institute of AI and Informatics in Medicine, School of Medicine, Klinikum Rechts der Isar, Technical University of Munich, 81675 Munich, Germany; bernhard.haller@tum.de (B.H.); cornelia.fuetterer@tum.de (C.F.)

**Keywords:** COVID-19, infodemic, health-care workers, HCW, information strategies, emergency information

## Abstract

Background: The COVID-19 pandemic has led to a flood of—often contradictory—evidence. HCWs had to develop strategies to locate information that supported their work. We investigated the information-seeking of different HCW groups in Germany. Methods: In December 2020, we conducted online surveys on COVID-19 information sources, strategies, assigned trustworthiness, and barriers—and in February 2021, on COVID-19 vaccination information sources. Results were analyzed descriptively; group comparisons were performed using χ^2^-tests. Results: For general COVID-19-related medical information (413 participants), non-physicians most often selected official websites (57%), TV (57%), and e-mail/newsletters (46%) as preferred information sources—physicians chose official websites (63%), e-mail/newsletters (56%), and professional journals (55%). Non-physician HCWs used Facebook/YouTube more frequently. The main barriers were insufficient time and access issues. Non-physicians chose abstracts (66%), videos (45%), and webinars (40%) as preferred information strategy; physicians: overviews with algorithms (66%), abstracts (62%), webinars (48%). Information seeking on COVID-19 vaccination (2700 participants) was quite similar, however, with newspapers being more often used by non-physicians (63%) vs. physician HCWs (70%). Conclusion: Non-physician HCWs more often consulted public information sources. Employers/institutions should ensure the supply of professional, targeted COVID-19 information for different HCW groups.

## 1. Introduction

To date, the COVID-19 pandemic has cost approximately 6.7 million lives [1] and had a major impact on health-care systems [2]. It has affected the lives of billions of people worldwide [3,4]. However, as the current SARS-CoV-2 variants less often seem to cause severe disease outcomes [5,6], countries are aiming to re-establish a “new normal” [7]. Despite these efforts, the pandemic is still ongoing, causing fatalities around the globe [1].

With the onset of the pandemic, the amount of information available on the etiology, epidemiology, prevention, and treatment of COVID-19 constantly increased. The growing body of evidence on COVID-19-related information is increasingly leading to an information overload, meaning that so much potentially relevant information is available that “it becomes a hindrance rather than a help” [8,9,10]. The World Health Organization (WHO) is now addressing the “infodemic” [11] as a problem. While this problem affects the general population [9], it imposes a significant barrier to health-care workers, where the overload of COVID-19-related information has been shown to be negatively associated with systemic information processing [12].

According to the WHO (taking into account the International Standard Classification of Occupations 2008 [13]), health workers—in this manuscript named health-care workers (HCWs)—can be defined as health professionals (e.g., medical doctors, nursing professionals, pharmacists, etc.), health associate professionals (e.g., technicians, assistants, etc.), personal-care workers in health services (e.g., health-care assistants, etc.), health management and support personnel (e.g., health-service managers, etc.), and health-service providers not elsewhere classified (e.g., armed forces occupations, other health service providers, etc.) [14]. In this study, we will focus on non-physician and physician health-care workers working either in a hospital, a doctor’s practice, or a public health facility.

To make the flood of information digestible, numerous dissemination approaches have been implemented targeted at HCWs [15,16]. Dissemination is defined as “the targeted distribution of information and intervention materials to a specific public health or clinical practice audience” [17]. These dissemination strategies need to be tailored to meet the needs and preferences of specific populations that should receive certain information by combining information-seeking and communication models [18]. However, these populations expose varying information-seeking behaviors, which are commonly defined as “the purposive seeking for information as a consequence of a need to satisfy some goal” [19]. Furthermore, the “information-seeking behavior of physicians and nurses is “[…] defined as the way physicians and nurses search for and utilize information to satisfy that information need” [20]. Data from our study on the use of COVID-19-related information among intensive care staff already showed that different information-seeking behavior was present among physicians and nurses in this particular staff group [21,22], indicating the need for targeted and tailored information.

In light of the COVID-19 pandemic, CEOsys, a research group of German university hospitals, was launched in 2020, supported by the German federal Ministry of Education and Research [23]. CEOsys aimed at creating tailored evidence syntheses on COVID-19 for German-speaking professionals and non-professionals. However, creating evidence syntheses was not the only mission of the network, but also developing strategies to improve the translation of the created scientific knowledge into policy and practice. To support these efforts, it was our objective to explore the information-seeking behavior of HCWs in light of decisions regarding COVID-19. In particular, in our Study 1, we were interested in exploring which information sources, channels, and formats HCWs used or preferred for seeking COVID-19-related information. We also wanted to explore which sources were regarded as particularly trustworthy and what barriers hindered non-physician and physician HCWs in patient care and non-patient care settings from seeking information from evidence syntheses. Additionally, we wanted to explore physician and non-physician HCWs’ preferred channels to obtain information on the COVID-19 vaccination by using data from our COVID-19 Vaccination Acceptance and Hesitancy study of HCW in Germany [24,25] (Study 2).

With the results, we would like to contribute to establishing better information pathways for the different professional HCWs’ groups to better prepare communication not only for further pandemic waves and challenges but also for daily routine.

## 2. Materials and Methods

We adhered to the STROBE checklist on reporting cross-sectional studies in writing this manuscript [26].

### 2.1. Surveys

We drew upon data collected with two separately administered surveys, the COVID-19 Informational Needs and Strategies Study (Study 1) and the COVID-19 Vaccination Acceptance and Hesitance Study (Study 2) [24,25]. In Study 1, the informational needs and strategies of HCWs regarding COVID-19 were queried. The study consisted of two surveys: One survey focused on HCWs in hospitals and doctor’s practices (i.e., including physicians, nursing staff, and other non-physician medical professionals). The other survey focused on HCWs in public health institutions (i.e., administratively and practically working physicians, and non-physicians). Study 2 focused on HCWs’ vaccination acceptance and hesitancy and also on preferred information channels regarding COVID-19 [24,25]. In this manuscript, from Study 2, results from physicians, nursing staff, other non-physician medical staff, dentists, and non-physician dentistry staff will be presented.

#### 2.1.1. COVID-19 Informational Needs and Strategies Study (Study 1)

We conducted a cross-sectional, exploratory study [27] using data collected via two voluntary open online surveys (one for hospitals and doctor’s practices and one for public health services) facilitated by SoSci Survey [28]. The surveys were part of a project on COVID-19 information seeking in different target groups conducted by members of the research group CEOsys [21,22,23,29]. Both surveys contained an electronic questionnaire in German that was distributed via e-mail. The surveys were open from 3 to 31 December 2020. One reminder was sent out after two weeks. For data protection reasons, personalized links were not sent to avoid traceability. Due to the urgency, a classic pen-and-paper survey was not possible, nor was it feasible given the second pandemic wave running in Germany at the time [30]. In addition, the snowball sampling method [31] was intended to facilitate the forwarding of the link to other members of the respective organization. No further advertisement was applied, and no incentives were provided.

##### Survey Participants (Study 1)

**Figure 1:**
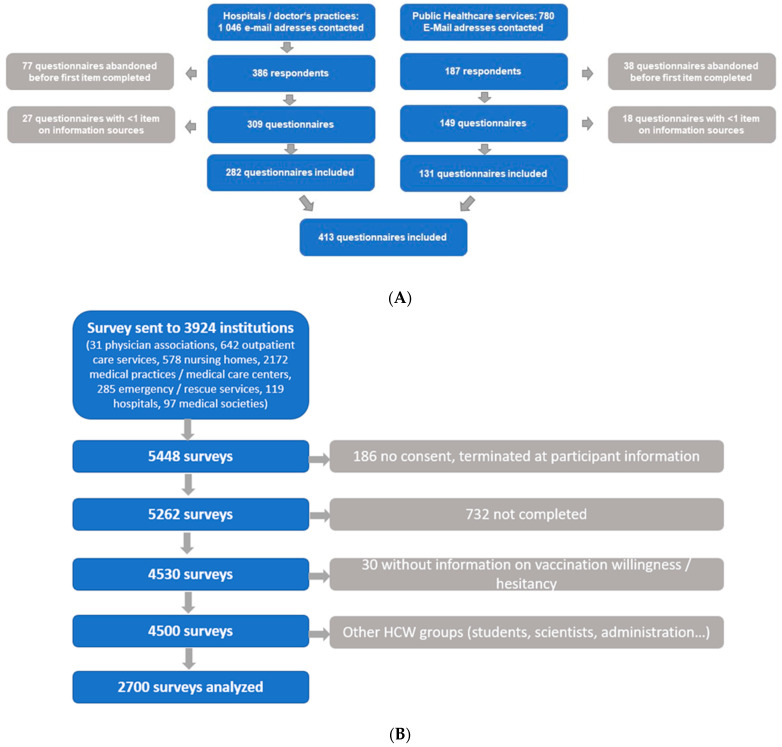
Flow Chart—Participation. (**A**) COVID-19 Informational Needs and Strategies Surveys (Study 1) (**B**) COVID-19 Vaccination Acceptance and Hesitancy Study (Study 2). Modified from Holzmann-Littig et al. [24,25].

In the survey on HCWs in hospitals and doctor’s practices, the link to the survey was sent to 1046 e-mail addresses of staff in physician practices, medical care centers, and hospitals in Bavaria (greater region of Munich, region of Wuerzburg) and Saxony (region of Leipzig). E-mail addresses of physician practices and medical care centers were taken from a public physician registry containing 8776 registered doctors. All available e-mail addresses not containing any names of the persons contacted were used (Figure 1).

For the survey on HCWs in public health organizations, we defined any entity as eligible that provides public health services with the aim to protect, restore, promote, and improve the health of populations [32]. The branch offices of all associations of statutory health insurance doctors, all health offices, district offices, and district administrations (depending on the local responsibility for the health sector), all county councils, all state medical associations, health insurance funds, nursing support centers, health ministries, associations of panel dentists, German Hospital Association and physicians’ unions, of which a general e-mail address could be found after searching the public telephone directories and registers, were contacted. Eventually, the survey link was sent to 780 non-personalized e-mail addresses (Figure 1).

##### Questionnaire (Study 1)

The questionnaires consisted of 14 questions in the survey in hospital and doctor’s practices staff and 15 in public-health-care staff, respectively, presented on 8 different screens. The questions were designed as closed questions with single- and multiple-choice answer options. Free text answer options were provided. Adaptive questioning was used for demographic items and to specify preferred information strategies. No review steps were provided. Questions on the professional setting, informational needs during the COVID-19 pandemic, preferred ways of COVID-19-related knowledge acquisition, perceived barriers to knowledge acquisition from evidence syntheses, information sources regarded as trustworthy, and (additionally, in the public health panel) strategies to determining a source’s trustworthiness were included. Since questions were asked in these surveys that could allow conclusions to be drawn about the work behavior or even political opinions of the participants, no question was asked about the age group or gender to exclude traceability with absolute certainty. The full questionnaire can be found in Supplemental Table S1. Since—to the best of our knowledge—no directly comparable surveys have already been conducted at that time of the pandemic, we constructed our own survey questions. The questions were developed by our working group within the framework of the CEOsys project and used in adapted form in each case for surveying different target groups, including our survey of intensive care staff [21,22] and laity [29]. Regarding preferred dissemination platforms and channels, we selected those known to provide COVID-19-related information independent of the accuracy of the information. Barriers were selected based on the recent scientific literature [33,34,35,36,37]. GESIS—Leibniz Institute for the Social Sciences survey guidelines for question-wording were considered [38]. The questionnaire was reviewed and pretested by members of the CEOsys network and authors’ colleagues in the respective departments. The items were not randomized within the questionnaires. We took account of the Checklist for Reporting Results of Internet E-Surveys (CHERRIES) [39] (Supplemental Table S2) to give readers a better understanding of the sample.

#### 2.1.2. COVID-19 Vaccination Acceptance and Hesitance Study (Study 2)

To compare the survey results with a larger dataset, we used data from our COVID-19 Vaccination Acceptance and Hesitancy Study [24,25]. In this study, data on media usage to gather information on the COVID-19 vaccines and trust in regulatory authorities were also collected; the full methodology can be found in the respective publications by Holzmann-Littig et al. [24,25]. This study was also performed as part of the CEOsys project [23].

##### Participants

The dataset was collected in a voluntary open online survey conducted from 2 February 2021 to 28 February 2021 in German language, using a cross-sectional exploratory [27] study design [24,25]. The survey link was sent to a total of 3924 e-mail addresses of nursing homes, medical practices, ambulance services, medical universities, hospitals, ambulatory care services, and medical societies across Germany [24,25]. One reminder was sent out after two weeks. All e-mail addresses were taken from publicly accessible hospital registries, online telephone books, and online physician registries for all participant groups in each German federal state. Again, recipients were asked to forward the link within their institution (snowball sampling [31]).

##### Questionnaire

The original questionnaire included 54 items. The complete question set that included several items on vaccine acceptance and hesitancy can be found in the supplement of Holzmann-Littig et al. [24]. No review steps were provided. For the present study, the items on feeling well informed on vaccines and trust in authorities (5-point Likert scales) were analyzed (combining the categories “fully agree” and “rather agree” to “agree” and the categories “I do not agree at all”, “I rather do not agree” and “I neither agree nor disagree” to “disagreement and indecisive”). Furthermore, the item on media usage (multiple answers possible) was analyzed. Within these items, no adaptive questioning was applied. For reasons of comparability, only participants in the groups “medical staff” and “non-physician medical staff” were included. All other groups, such as students and administrative staff, were excluded from the analysis. The items were not randomized within the questionnaires.

### 2.2. Ethics and Data Protection

For Study 1, ethics approval was obtained from the Institutional Review Board of the Medical Faculty at the University of Würzburg (reference number 2020-219/20). For Study 2, approval from local ethics committees (41/21 S), data protection officers, hospital boards, and staff councils was obtained. All European, German, and local data protection requirements were followed. Every participant gave informed consent by clicking a checkbox prior to completing the survey. For data protection reasons, no IP addresses or view rates were recorded, no cookies were used, no log file analysis was applied, and no participant registration was demanded in any survey. Additionally, no e-mail addresses containing personal information were selected. The study adheres to the Declaration of Helsinki.

### 2.3. Statistics

Statistical analysis was performed using R, version 4.1.2 (R Foundation for Statistical Computing, Vienna, Austria) and Microsoft^®^ Excel^®^. All data are presented as absolute and relative frequencies. For group comparisons of the different application areas (e.g., of physicians vs. non-physicians as well as patient care vs. non-patient care), odds ratios with corresponding 95% confidence intervals are presented. Furthermore, two-sided χ^2^-tests were conducted based on a significance level of 5%. Missing answers were excluded per item from analysis, the number of missing data per item is indicated in Supplemental Table S3. Professional groups were grouped based on the respondents’ answer to the respective multiple-choice question as “physician”/“non-physician” and “patient care”/“non-patient care” in Study 1. If the answer option “other” was selected respondents were assigned to the respective groups based on their free text comment. Due to the study’s exploratory nature, no adjustment for multiple testing was undertaken. Only questionnaires with at least one completed question subset were analyzed. Because it is possible that the surveys were conducted at the workplace, which can lead to interruptions and highly divergent response times, the analysis of atypical time stamps was omitted.

## 3. Results

### 3.1. Participants

In Study 1, the survey links were sent to a total of 1826 e-mail addresses, with 573 responses. Of these, 115 were abandoned directly after opening. Another 45 questionnaires contained answers to <1 item on information sources, and therefore, also had to be excluded. Consequently, 413 questionnaires could be included in the analysis (see Figure 1). Of these, 299 participants (72.4%) were physicians, and 114 (27.6%) were non-physician medical staff. An amount of 303 participants (73.4%) were involved in patient care, and 110 (26.6%) were not involved in patient care. Of HCWs working in patient care, 23.4% (71/303) were non-physician HCWs, and in the group of HCWs not working in patient care, 39.1% (43/110) were non-physician HCWs, *p* = 0.002.

In Study 2, the survey link was sent to 3924 e-mail addresses. Of the formerly analyzed 4500 questionnaires, 2700 could be categorized to either physician or non-physician HCWs, and therefore, could be included in this analysis. An amount of 859 participants (31.8%) were non-physician HCWs (Figure 1).

Participants’ professions are listed in more detail in Table 1.

Due to the snowball sampling method (i.e., forwarding the link within the respective institution), the number of recipients of the link—and accordingly, response rates—cannot be calculated.

### 3.2. COVID-19 Information Needs and Strategies Study (Study 1)

#### 3.2.1. Information Channels

##### Social Media

Regarding social media as a channel for COVID-19 information (multiple choice question) for physicians, research portals, such as ResearchGate^®^, were most important, whereas non-physicians most often selected Facebook^®^ in this category. Facebook, Instagram^®^, and YouTube^®^ were significantly less often used by physicians compared to non-physicians, whereas research portals were significantly more often used by physicians. All absolute and relative frequencies, as well as Odds ratios and confidence intervals are presented in Table 2.

**Figure 2:**
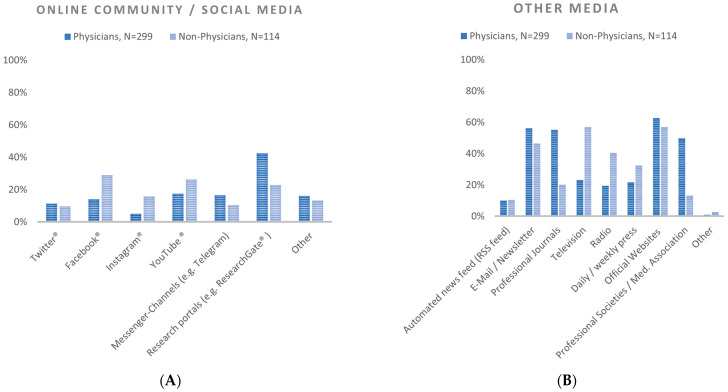
Media usage in physicians and non-physicians, relative frequencies. (**A**) Online community/social media, (**B**) other media.

**Figure 3:**
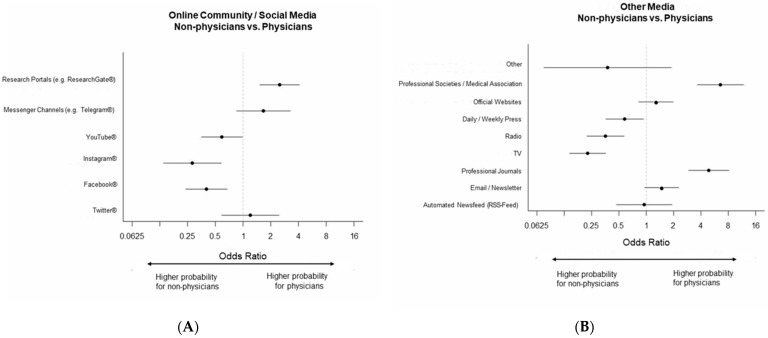

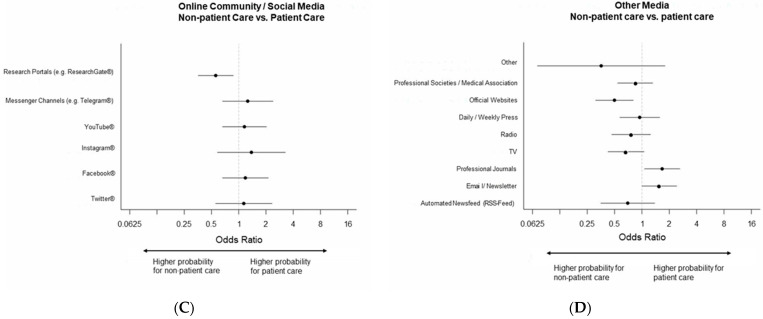
Media usage, Forest plots (**A**) Odds ratios of survey category Online community/social media, non-physicians vs. physicians, (**B**) Other media, non-physicians vs. physicians, (**C**) Online community/social media, non-patient care vs. patient care, (**D**) Other media, non-patient care vs. patient care.

Regarding the professional setting (patient care vs. non-patient care), a significant difference was only found for research portals, with personnel involved in patient care choosing this item less often than personnel not involved in patient care (Figure 2 and Figure 3, Table 2 and  Supplemental Table S3).

##### Other Media

In this multiple-choice question, physicians selected official websites most often. Non-physicians selected official websites and television, both most often. Professional journals and professional societies/medical associations were both selected significantly more often by physicians. Non-physicians selected TV, radio and daily/weekly press significantly more often than physicians (Figure 2 and Figure 3, Table 2 and Supplemental Table S3).

#### 3.2.2. Information Sources Regarded as Trustworthy

When asked the multiple-choice question: “Which organizations (source of information) do you consider to be particularly reliable with regard to information on medical therapy for COVID-19?” the German Robert-Koch-Institute was chosen most often by far in both groups with non-physicians significantly more often choosing this item. However, further significant differences between non-physician HCWs and physicians were observed. Non-physicians significantly more often considered the World Health Organization and the German Federal Center for Health Education German: BZgA, Bundeszentrale für Gesundheitliche Aufklärung as particularly reliable regarding information on medical therapy for COVID-19.

**Figure 4:**
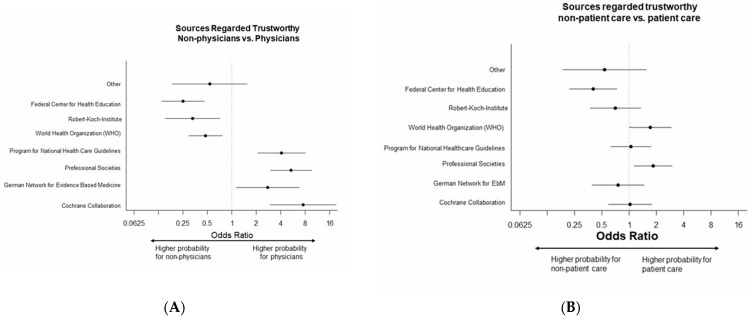
Sources of Information regarded as particularly trustworthy for information on medical treatment of COVID-19, Forest plots, Odds ratios for, (**A**) non-physicians vs. physicians, (**B**) non-patient care vs. patient care.

In contrast, physicians significantly more often chose the Cochrane Collaboration, the German Network for Evidence Based Medicine, Professional Societies and the Program for National Health Care Guidelines. Supplemental Figure S1 and Figure 4, all absolute and relative frequencies, Odds ratios, and corresponding confidence intervals are presented in Table 3 and Supplemental Table S3.

Only a few significant differences were observed regarding the work setting, i.e., patient care vs. non-patient care. Professional societies were selected significantly more often by staff working in patient care, the latter was observed for the World Health Organization. The German Federal Center for Health Education was selected more often by non-patient care staff (Figure 4, Table 3 and Supplemental Table S3).

#### 3.2.3. Barriers to Using Evidence Syntheses

Asked for barriers to the use of evidence syntheses on COVID-19 (multiple-choice question), lack of time was chosen most often by far. For non-physicians, the second most often selected barrier was lack of experience. The second most often selected item for physicians was access barriers (e.g., financial aspects). The third most often selected item in both groups was how to access the information (Table 4, Supplemental Table S3, Supplemental Figure S2).

#### 3.2.4. Information Formats

Participants were also asked about their preferred information formats (multiple choice question): “Particularly with regard to the dynamically developing evidence and knowledge situation during a pandemic, which strategies are the most effective for you to gain knowledge of specific content and put it into practice?”

##### Online/Print Information Materials (in Professional Websites, Apps, Journals)

**Figure 5:**
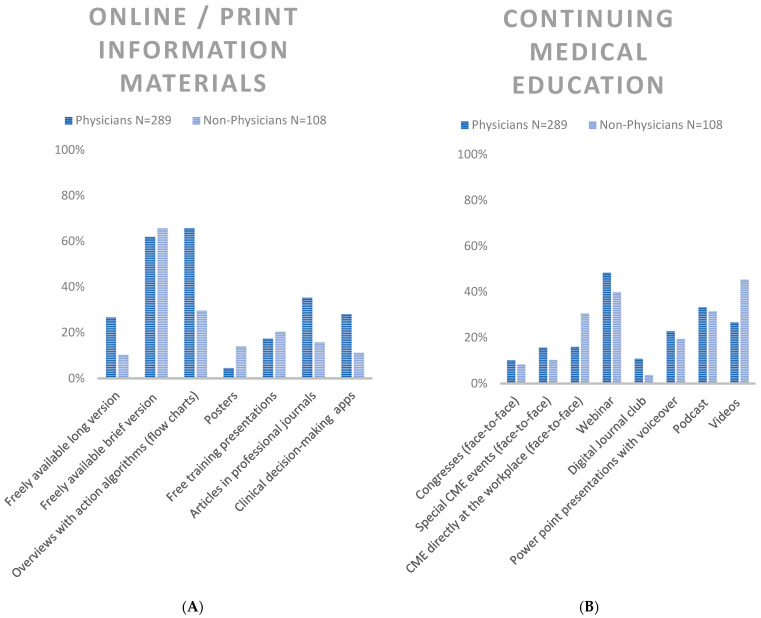
(**A**) Preferred Information Formats Online/and Print Media, relative frequencies, (**B**) Continuing Medical Education, relative frequencies; both for physicians and non-physicians.

**Figure 6:**
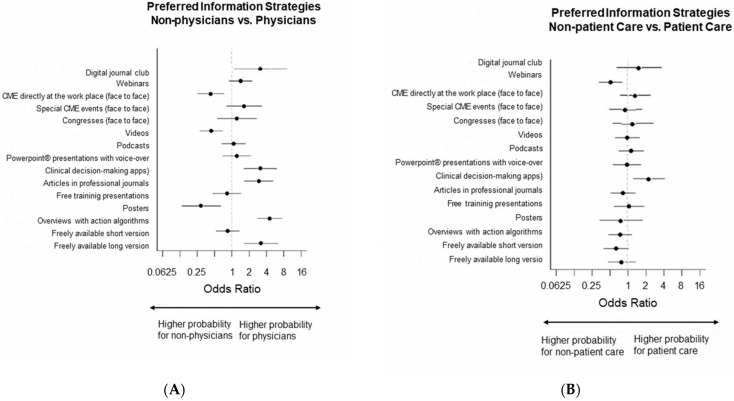
Preferred Information Formats, Forest plots, Odds ratios for (**A**) non-physicians vs. physicians, (**B**) non-patient care vs. patient care.

For non-physicians freely available brief versions were the most and for physicians the second most appreciated information materials (non-physicians: 65.7%, 71/108; physicians: 61.9, N = 179/289, *p* = 0.485). Physicians selected overviews with action algorithms most often, non-physicians chose this item second most often: (non-physicians: 29.6%; 32/108, physicians 65.7%, 190/289, *p* < 0.001 (Figure 5 and Figure 6, Supplemental Table S3).

##### Continuing Medical Education

Regarding Continuing Medical Education (CME), videos were most popular in non-physicians (45.4%, 49/108); however, physicians selected this item significantly less often with 26.6%, 77/289, *p* < 0.001. Webinars were most popular in physicians (48.4%, 140/289) and second most popular in non-physicians (39.8%, 43/108), *p* = 0.125. Podcasts were second most popular in physicians (33.2%, 96/289) and third most popular in non-physicians (31.5%, 34/108), *p* = 0.743 (Figure 5 and Figure 6, Supplemental Table S3).

### 3.3. COVID-19 Vaccination Acceptance and Hesitancy Study (Study 2)

#### 3.3.1. Media Usage

To compare our results with results from a larger cohort, we used data from Study 2. In this cohort, for physicians (choosing this item significantly more often than non-physicians), a major source of information was professional journals. The second most often selected item for physicians was official websites (e.g., by the German Robert Koch Institute, the World Health Organization, etc.). Interestingly, while for gathering COVID-19-related information in general, in our first cohort newspapers played a minor role for physicians; in this cohort, newspapers were named third most often by physicians. Non-physicians used official websites most often, followed by TV/Radio and Newspapers. Table 5, Supplemental Figure S3.

#### 3.3.2. COVID-19 Knowledge Test Results and Media Usage

Furthermore, participants were asked to answer a knowledge test on the COVID-19 vaccination. The questions can be found in Supplemental Table S1. In median, participants reached 3 out of 4 points in the knowledge test.

**Figure 7:**
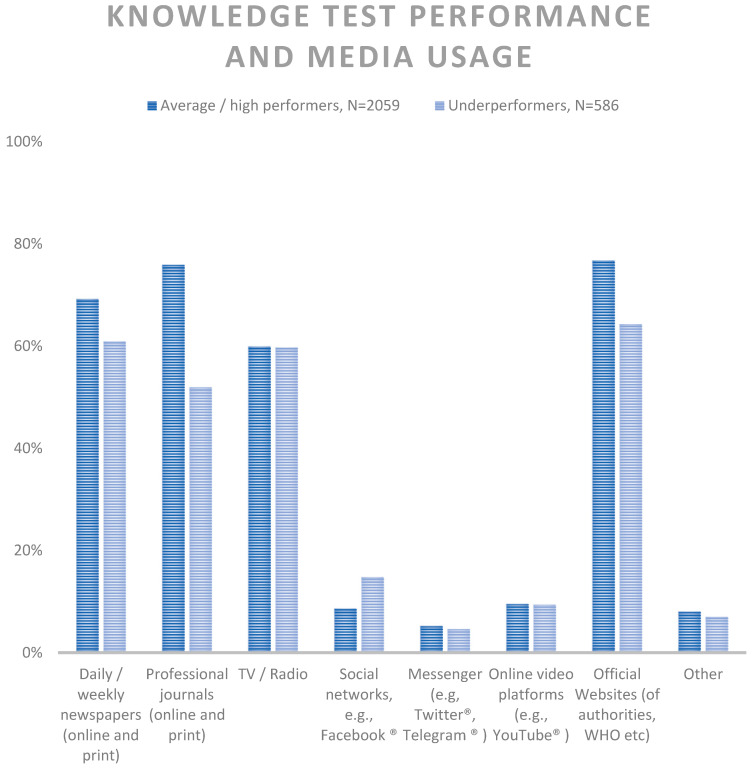
Knowledge test performer status and media usage, relative frequencies.

Significant differences were observed in the media usage of underperformers (<3 points) compared to average/high performers (3–4 points). Notably, underperformers used official websites (average/high: 76.8% (1581/2059) vs. low 64.3% (377/586), *p* < 0.001), professional journals (average/high: 75.9 (1562/2059) vs. low: 51.9 (304/586), *p* < 0.001) and newspapers (average/high: 69.2, (1425/2059) vs. low 60.9 (357/586), *p* < 0.001) significantly less often than average and high performers (Figure 7).

#### 3.3.3. Trust in Authorities

As the results from Study 1 showed that most respondents used official websites (e.g., by the German Robert-Koch Institute), indicating trust in official/authority information sources, we analyzed the items on trust in authorities in the larger cohort. In the COVID-19 vaccine acceptance and hesitance study, trust in authorities was relatively high, with a higher percentage of physicians stating to trust authorities. In total, 90.0% of physicians agreed or fully agreed to trust the regulatory authorities of vaccines in Germany in general; in non-physicians, this was the case in 85.0%; *p* < 0.001. Trust in European regulatory authorities was slightly lower, as 83.1% of physicians and 78.6% of non-physicians agreed or fully agreed with the statement “I trust the European regulatory authorities of COVID-19 vaccines used in Germany”; *p* < 0.001. A very different picture was observed when asking for trust in German health politics, 58.8% of physicians and 56.7% of non-physicians agreed or fully agreed with the statement “I generally trust German health care politics”; *p* = 0.299 (Table 5).

## 4. Discussion

Seeking and locating relevant and useful information on COVID-19 was reported to be challenging for different groups of HCWs, particularly during an infodemic.

Better communication to the different groups of HCWs in their different fields of application is still challenging with the increase of information. HCWs in hospitals, practices, and public health authorities consulted a wide range of dissemination channels when seeking relevant information for their daily decision-making. As in our previous studies among intensive care professionals [21,22], this study revealed substantial differences in the use of information sources on COVID-19 between non-physician and physician HCWs. Interestingly, fewer significant differences were found between HCWs in patient care and non-patient care (i.e., public health).

### 4.1. Information Channels

The HCWs included in our study consulted various scientific and public sources of information. Non-physician HCWs used publicly available media such as TV besides official websites and newsletters as one of their primary sources of information about COVID-19, but the radio was also frequently used. However, in a recent study, only about 19% of HCWs in Germany trusted media regarding COVID-19 [40]. Even more, it appears that non-physician HCWs might have depended on public media to receive information on COVID-19. On the other hand, physicians’ most important sources of information were official websites, newsletters, and professional journals. Facebook^®^, YouTube^®^, and Instagram^®^ were also significantly more frequently used by non-physician HCWs than physician HCWs. A recent study evaluated YouTube^®^ as a source of medical information on the COVID-19 pandemic and rated 69.9% (79 videos) as useful, i.e., containing scientifically correct information [41]. However, if providing professional information for HCWs on this platform is beneficial remains to be investigated. Physicians seem to use professional journals far more often than non-physicians, which is in line with Study 2. A major source of information for physicians was professional journals (online and print), whereas non-physicians consulted this source of information less. Overall, consistent with the results from our studies in intensive care staff [21,22], it can be seen that physician HCWs accessed professional, scientific sources to learn about COVID-19 significantly more than non-physician HCWs.

Interestingly, while newspapers played a minor role for physicians gathering COVID-19-related information in general in our first cohort, in Study 2, when seeking information on the COVID-19 vaccination, newspapers were named third most often by physicians (69.5%). This might be due to the fact that very recent developments on COVID-19 vaccines were discussed daily in the newspapers in Germany at that time, and it was an easy way to gather this information. Non-physicians used official websites most often, however, again followed by TV and radio. In a Finnish study, nursing staff also reported using traditional and social media, but also real-life social networks, including friends and family, as main sources of information on COVID-19 vaccines [42].

This suggests that the information needs of non-physician HCWs might not have been adequately addressed, and thus, non-physician HCWs might have been disadvantaged in the dissemination of scientific and professional information on COVID-19. In addition, in a study conducted in 2020, nurses reported a “need for […] education and training to provide care for patients with COVID-19” [43]. In a recent study, a positive perception of quality and safety of care was regarded as a pull factor, i.e., a factor contributing to not quitting the job for nursing staff [44]. Furthermore, a study from Poland showed that besides financial stability, the flow of information was a significant predictor of WHO-5, PHQ-9, and ISI scores [45]. According to Wan and Xia, “It is crucial to accumulate, bind, and effectively utilize human resources” [46]. However, in a recent study, “occupational stress was more closely related to perceptions of lack of distributive justice than to perceptions of procedural, informational, and interpersonal justice” [47]. However, the authors also discussed that a “lack of correctness […] in the transmission of the information necessary for the work (informational justice) could interfere with the diagnostic and therapeutic procedures that take place in the hospital.” [47] Having sufficient professional information may be regarded as substantial to provide high-quality care—so this must be taken into account concerning the background of the known problem of nursing staff shortages [48,49]. Especially in times when staff need to be moved quickly from one ward to another, proper provision of information is essential [50]. In addition, lack of information appears to be associated with vaccine hesitancy [51] and must therefore be avoided.

### 4.2. Information Sources Regarded as Trustworthy

Strikingly, the German Robert Koch Institute was by far considered the most trusted source for COVID-19 information among both physician and non-physician HCWs. This is in line with a recent study that also identified the Robert Koch Institute as the institution most often regarded as trustworthy by German HCWs in terms of COVID-19-related information [40]. This appears relevant, considering that trust in the health-care system, for example, is an essential precondition for the acceptance of vaccinations [52], and vaccine hesitant HCWs are more often mistrustful toward authorities [24,53]. Therefore, it appears of utmost importance that these authorities provide professional medical targeted information for HCW. However, there seems to be a major difference in the perception of health authorities in Germany and health care politics on the other hand. Here, both, physicians and non-physicians showed much lesser trust, with no significant difference between both groups.

### 4.3. Information Formats

Looking at the most frequently mentioned preferred information formats, formats providing concise scientific evidence in a user-friendly format could be very helpful for disseminating information to physician and non-physician HCWs. This might be due to the lack of time mentioned by participants and described in the literature [34,35]. Therefore, brief versions should be made available for both audiences. It is also consistent with the fact that both groups indicated that they consider newsletters an essential source of information since these often offer condensed information.

In case of other pandemic events, however, the overviews with action algorithms required by physician HCWs could also be developed and disseminated at an early stage. The production of informational videos, which has been requested more often by non-physician HCWs, should also be considered at an early stage, even though this involves considerable effort. Both non-medical and medical HCWs demanded webinars which are also discussed in the literature for continuing medical education [54]. This should also be planned early in the event of renewed pandemic events or other waves. However, as stakeholder involvement is also discussed in the literature [55], more inclusion of the target groups into creating and disseminating scientific and professional content might be beneficial too.

### 4.4. Information Barriers

The main barrier to using evidence synthesis was the lack of time. This problem is described in the literature [34,35,56] and persists. It seems necessary to provide HCWs with the possibility to quickly obtain professional information, be it via information portals with summaries and action algorithms as well as videos or via newsletters. There were also access problems, e.g., with paid accounts. This problem is described in the literature [36,37]—it may be counteracted by the growing number of Open Access Journals [57]. However, state institutions and clinics should also ensure that their employees can access information. Furthermore, our results on barriers appear to be in line with the literature, as Aakre et al. found “time”, “resource accessibility”, “personal attitudes and information-seeking skills”, “institutional attitudes, cultures and policies” as well as “knowledge resource features” as determinants to information seeking [58].

### 4.5. Strengths and Limitations

This study has a couple of strengths. First, it is a study on different groups of HCWs, not only in hospitals and doctor’s practices but also in public health institutions. We are therefore providing insights into HCWs’ information seeking behavior in different work settings and for different types of HCWs. By this, we are trying to support optimal information provision in (but not limited to) other crisis or pandemic situations. In addition, on the one hand, asking for primary sources of information and organizations regarded as trustworthy and, on the other, for preferred information formats, we were able to provide quite a comprehensive picture of possible ways to improve information flow to HCWs. Furthermore, we could compare some of the results in a larger cohort two months later, underlining our findings.

However, this study has weaknesses that need to be addressed. First, in Study 1, we used a small, non-representative sample with physicians and non-physicians in the hospital and doctor’s practice setting in Bavaria (greater region of Munich, region of Wuerzburg) and Saxony (region of Leipzig). In addition, in the public health cohort in this study, the study sample may not be fully representative, although a wide range of organizations were contacted. However, we therefore compared some of our results to those from the larger cohort of Study 2, leading to mostly comparable results. Furthermore, we do not have data on participants’ sexes in Study 1. At that time, surveys on opinions regarding COVID-19 were susceptible and traceability to individuals had to be ruled out by any means. Thus, although some results are confirmed in the larger cohort of Study 2, generalizability may be somewhat limited. We therefore recommend more extensive international studies on information-seeking behavior of HCWs in general, also including data on age and gender. Moreover, online surveys may be beneficial to achieve comprehensive responses in short periods. However, this is opposed to classic pen-and-paper designs and careful selection of a representative sample, which would have taken far too much time as data were urgently needed for the CEOsys network to create tailored information materials [59]. Furthermore, an online survey appeared to be advisable considering the necessity of reducing interpersonal contact. However, as all surveys were constructed as open online surveys, it is impossible to estimate how representative the sample is, and response rate calculation is impossible. A primary goal was to reach as many HCWs as possible, so the method of snowball sampling [31] was applied. Therefore, it could not be foreseen how the link would be shared and how many HCWs would be willing to participate, and no formal sample size calculation was performed. Furthermore, selection bias is a known issue in online surveys [60] and cannot be ruled out. Consequently, it might be possible that users of online information sources are overrepresented in this study. Further studies should address this issue with at least additional pen-and-paper surveys for HCWs’ groups with infrequent computer access.

Moreover, our studies have considered only a selection of possible sources of information as they should be used to find optimal dissemination strategies for the CEOsys network. However, according to Hurst and Mickan, “six categories of knowledge encounters” exist with corresponding facets [61]. In addition, consultation with colleagues may be an important source of information [62], which has not been examined in this manuscript. Further research should also take these facets of information-seeking behavior into account.

Furthermore, the participant groups are not fully comparable between both studies; for example, in Study 1, non-physicians and physicians from non-patient care settings were involved, whereas in Study 2 participants only came from patient-care settings. However, since we did not find many significant differences between patient care and non-patient care settings, we believe that the results can still be compared to the data from Study 1.

## 5. Conclusions

Future efforts to disseminate scientific knowledge should make sure that information is targeted at and tailored to the respective HCWs’ group. Non-physician HCWs were significantly less likely to use professional sources of information and significantly more likely to use traditional media, such as television or radio, as well as social media, to find out about COVID-19. Therefore, relevant medical information should be offered to this group, particularly in a targeted manner, considering their preferred information channels and formats (summaries, videos, and newsletters). All relevant stakeholders should be included in the creation, dissemination, and knowledge translation of scientific information. Additionally, training on finding the correct answers to practice-relevant questions could be helpful for all HCWs’ groups. Providing information in concise and user-friendly formats such as action algorithms, summaries, and newsletters might be beneficial for physicians. Webinars could be a valuable complement for both groups. Employers should consider this, as should governmental institutions (which are highly trusted by HCWs in Germany, according to our data) when planning the dissemination of crucial medical information for HCWs. Since both groups frequently reported using official websites as a source of information, it may be helpful to build medical professional information portals that consider the different requirements of the two groups. Under no circumstances should any groups of HCWs be cut off from relevant information by insufficient adaptation of information strategies, as they are on the frontline, especially during pandemic situations, and need the best possible information, not least to exert a positive influence on the patients and their influence through their example.

## Figures and Tables

**Table 1:** Participants’ professions.

Study 1				Study 2	
Hospitals/Doctor’s Practices		Public Health Care Services		Inpatient and Outpatient Care, N = 2700	
Physicians, N = 211	Non-physicians, N = 71	Physicians, N = 88	Non-Physicians, N = 43	Physicians, N = 1841	Non-Physicians, N = 859
Residents, N = 91 Specialist physicians, N = 120	Nursing staff, N = 45 Other non-physician medical professionals, e.g., medical technical assistants, N = 26	Physicians, practically active, N = 42 Physicians, administra-tively active, N = 46	Non-physician health care professionals, hands-on, N = 5 Non-physician health care professional, administrative, N = 6 Other function in administration/organization, N = 23 Other, N = 9	Residents, N = 323 Specialist Physicians, N = 1329 Dentists, N = 189	Nursing staff, N = 466 Other non-physician medical professionals, e.g., medical technical assistants, N = 346 Dentistry non-physician medical personnel, N = 47

**Table 2:** Results of informational Needs and Strategies survey (study 1), grouped by physicians/non-physicians and patient care/non-patient care.

Study 1, N = 413								
Item	Physicians, N = 299; N (%)	Non-Physicians, N = 114; N (%)	*p*	Odds Ratio [95% CI]	Patient Care, N = 303; N (%)	Non-Patient Care, N = 110; N (%)	*p*	Odds Ratio [95% CI]
Which of the following platforms and channels do you or would you use to become aware of newly generated evidence/treatment recommendations								
Online Community or Social Media								
Missing	0	0			0	0		
Twitter	34 (11.4)	11 (9.6)	0.616	1.20 [0.59–2.46]	34 (11.2)	11 (10.0)	0.725	1.14 [0.55–2.33]
Facebook	42 (14.0)	33 (28.9)	<0.001	0.40 [0.24–0.67]	57 (18.8)	18 (16.4)	0.568	1.18 [0.66–2.12]
Instagram	15 (5.0)	18 (15.8)	<0.001	0.28 [0.14–0.58]	26 (8.6)	7 (6.4)	0.463	1.38 [0.58–3.28]
Youtube	52 (17.4)	30 (26.3)	0.042	0.59 [0.35–0.98]	62 (20.5)	20 (18.2)	0.608	1.16 [0.66–2.03]
Messenger channel (e.g., Telegram)	49 (16.4)	12 (10.5)	0.133	1.67 [0.85–3.26]	47 (15.5)	14 (12.7)	0.481	1.26 [0.66–2.03]
Networking in a research portal (e.g., ResearchGate)	127 (42.5)	26 (22.8)	<0.001	2.50 [1.52–4.10]	101 (33.3)	52 (47.3)	0.010	0.56 [0.36–0.87]
Other	48 (16.1)	15 (13.2)	0.464	1.26 [0.68–2.36]	48 (15.8)	15 (13.6)	0.582	1.19 [0.64–2.23]
**Campaigns or advertisements via**								
automated newsfeed (RSS feed)	30 (10.0)	12 (10.5)	0.882	0.95 [0.47–1.92]	28 (9.2)	14 (12.7)	0.300	0.70 [0.35–1.38]
Email/newsletter	168 (56.2)	53 (46.5)	0.077	1.48 [0.96–2.28]	171 (56.4)	50 (45.5)	0.048	1.55 [1.00–2.41]
Professional journals	165 (55.2)	23 (20.2)	<0.001	4.87 [2.92–8.12]	148 (48.8)	40 (36.4)	0.024	1.67 [1.07–2.62]
Television	69 (23.1)	65 (57.0)	<0.001	0.23 [0.14–0.36]	91 (30.0)	43 (49.1)	0.082	0.67 [0.42–1.05]
Radio	58 (19.4)	46 (40.4)	<0.001	0.36 [0.22–0.57]	72 (23.8)	32 (29.1)	0.270	0.76 [0.47–1.24]
Daily/weekly press	65 (21.7)	37 (32.5)	0.024	0.58 [0.36–0.93]	74 (24.4)	28 (25.5)	0.830	0.95 [0.57–1.56]
Internet pages of the Robert Koch Institute, AWMF, Federal Ministry of Health, etc.	188 (62.9)	65 (57.0)	0.275	1.28 [0.82–1.98]	173 (57.1)	80 (72.2)	0.004	0.50 [0.31–0.80]
Professional societies/medical associations	149 (49.8)	15 (13.2)	<0.001	6.56 [3.64–11.81]	117 (38.6)	47 (42.7)	0.450	0.84 [0.54–1.31]
Other	3 (1.0)	3 (2.6)	0.216	0.38 [0.07–1.89]	3 (1.0)	3 (2.7)	0.192	0.36 [0.07–1.79]

**Table 3:** Information Sources regarded as trustworthy, grouped by physicians/non-physicians and patient care/non-patient care; absolute and relative frequencies, Odds ratios.

Study 1, N = 413								
Item	Physicians, N = 299; N (%)	Non-Physicians, N = 114; N (%)	*p*	Odds Ratio [95% CI]	Patient Care, N = 303; N (%)	Non-Patient Care, N = 110; N (%)	*p*	Odds Ratio [95% CI]
Which organizations (source of information) do you rate as most trustworthy regarding information on COVID-19 medical therapy?								
Missing	19	11			23	7		
Cochrane Collaboration	78 (27.9)	5 (4.9)	<0.001	7.57 [2.97–19.29]	61 (21.8)	22 (21.4)	0.928	1.03 [0.59–1.78]
German Network for Evidence-Based Medicine (EbM Network)	41 (14.6)	6 (5.8)	0.020	2.77 [1.14–6.74]	32 (11.4)	15 (14.6)	0.407	0.76 [0.39–1.46]
Professional societies (e.g., DIVI, DGAI, DGIIN)	139 (49.6)	16 (15.5)	<0.001	5.36 [2.99–9.60]	124 (44.3)	31 (30.1)	0.012	1.85 [1.14–2.99]
Program for National Health Care Guidelines (cooperation of German Medical Association, KBV, AWMF)	92 (32.9)	11 (10.7)	<0.001	4.09 [2.09–8.02]	76 (27.1)	27 (26.2)	0.856	1.05 [0.63–1.75]
World Health Organization (WHO)	69 (24.6)	42 (40.8)	0.002	0.47 [0.29–0.77]	89 (31.8)	22 (21.4)	0.046	1.72 [1.01–2.93]
Robert Koch Institute	223 (79.6)	95 (92.2)	0.004	0.33 [0.15–0.72]	229 (81.8)	89 (86.4)	0.285	0.71 [0.37–1.34]
Federal Center for Health Education	24 (8.6)	28 (27.2)	<0.001	0.25 [0.14–0.46]	29 (10.4)	23 (22.3)	0.002	0.40 [0.22–0.73]
Other	9 (3.2)	6 (5.8)	0.243	0.54 [0.19–1.55]	9 (3.2)	6 (5.8)	0.243	0.54 [0.19–1.55]

**Table 4:** Information Barriers, grouped by physicians/non-physicians and patient care/non-patient care; absolute and relative frequencies, Odds ratios.

Study 1, N = 413								
Item	Physicians, N = 299; N (%)	Non-Physicians, N = 114; N (%)	*p*	Odds Ratio [95% CI]	Patient Care, N = 303; N (%)	Non-Patient Care, N = 110; N (%)	*p*	Odds Ratio [95% CI]
What barriers do you face to acquiring evidence-based knowledge								
Missing	23	14			27	10		
I have too little time in my daily work to deal with evidence syntheses.	218 (79.0)	55 (55.0)	<0.001	3.08 [1.89–5.01]	200 (72.5)	73 (73.0)	0.918	0.97 [0.58–1.63]
The independent acquisition of evidence-based knowledge is not encouraged by my superiors.	30 (10.9)	10 (10.0)	0.809	1.10 [0.52–2.34]	34 (12.3)	6 (6.0)	0.079	2.20 [0.89–5.41]
I have no experience in dealing with evidence syntheses.	28 (10.1)	30 (30.0)	<0.001	0.26 [0.15–0.47]	40 (14.5)	18 (18.0)	0.405	0.75 [0.41–1.39]
I am unsure/don’t know where or how to access reliable evidence syntheses.	53 (19.2)	21 (21.0)	0.699	0.89 [0.51–1.58]	61 (22.0)	13 (13.0)	0.050	1.90 [0.99–3.63]
Access to evidence syntheses is too cumbersome (e.g., paid subscriptions/memberships).	99 (35.9)	17 (17.0)	<0.001	2.73 [1.53–4.86]	81 (29.3)	35 (35.0)	0.294	0.77 [0.47–1.25]
The language in evidence syntheses is too complex and difficult for me to understand.	13 (4.7)	20 (20.0)	<0.001	0.20 [0.09–0.42]	21 (7.6)	12 (12.0)	0.184	0.60 [0.29–1.28]
The content in evidence syntheses is not well matched to my target audience (e.g., in terms of prior knowledge, relevance).	31 (11.2)	17 (17.0)	0.139	0.62 [0.33–1.17]	33 (12.0)	15 (15.0)	0.435	0.77 [0.40–1.49]
I reject the acquisition of knowledge from evidence syntheses, because in my opinion the contents are not practicable in everyday life (e.g. too undifferentiated, not applicable to the individual).	3 (1.1)	1 (1.0)	0.942	1.09 [0.11–10.58]	1 (0.4)	3 (3.0)	0.028	0.12 [0.01–1.14]
Other	11 (4.0)	3 (3.0)	0.656	1.342 [0.37–4.91]	13 (4.7)	1 (1.0)	0.093	4.89 [0.63–37.90]

**Table 5:** Results from (study 2), grouped by physicians / non-physicians, Reference for Odds ratios: non-physicians.

COVID-19 Vaccination Acceptance and Hesitancy Study				
	Physicians N = 1841	Non-Physicians N = 859	*p*	Odd’s Ratio [95% CI]
From what media do you get your information on COVID-19 vaccination, N = 2700				
Missing	0	0		
Daily newspapers, weekly magazines (print)	620 (33.7)	231 (26.9)	<0.001	1.38 [1.15–1.65]
Daily newspapers, weekly magazines (online)	996 (54.1)	405 (47.1)	<0.001	1.32 [1.12–1.55]
Daily/weekly newspapers and magazines (online and print)	1280 (69.5)	537 (62.5)	<0.001	1.37 [1.15–1.62]
Scientific journals (print)	599 (32.5)	108 (12.6)	<0.001	3.35 [2.68–4.20]
Scientific journals (online)	1269 (68.9)	406 (47.3)	<0.001	2.48 [2.10–2.92]
Scientific journals (print and online)	1449 (78.7)	439 (51.1)	<0.001	3.54 [2.97–4.21]
Television/radio	1077 (58.5)	540 (62.9)	0.031	0.83 [0.71–0.98]
Social networks (e.g., Facebook)	150 (8.1)	122 (14.2)	<0.001	0.54 [0.42–0.69]
Messenger services (e.g., Twitter, Telegram)	94 (5.1)	43 (5.0)	0.912	1.02 [0.71–1.48]
Online video platforms (e.g., YouTube)	157 (8.5)	100 (11.6)	0.010	0.71 [0.54–0.92}
Websites/information portals of government health authorities (e.g., RKI, WHO)	1346 (73.1)	649 (75.6)	0.179	0.88 [0.73–1.06]
Other	114 (6.2)	95 (11.1)	<0.001	0.53 [0.40–0.71]
**Knowledge test**				
Missing	20	35		
0 points	34 (1.9)	40 (4.9)	<0.001	0.37 [0.23–0.59]
1 point	62 (3.4)	71 (8.6)	<0.001	0.37 [0.26–0.53]
2 points	215 (11.8)	164 (19.9)	<0.001	0.54 [0.43–0.67]
3 points	485 (26.6)	299 (36.3)	<0.001	0.64 [0.53–0.76]
4 points	1025 (56.3)	250 (30.3)	<0.001	2.96 [2.48–3.52]
**I trust the regulatory authorities of vaccines in Germany in general**				
Missing	0	0		
I fully agree/I rather agree	1655 (90.0)	727 (85.0)	<0.001	1.62 [1.27–2.05]
**I trust the European regulatory authorities of COVID-19 vaccines used in Germany**				
Missing	0	0		
I fully agree/I rather agree	1530 (83.1)	670 (78.6)	<0.001	1.39 [1.13–1.70]
**I generally trust in the German health care politics**				
Missing	0	0		
I fully agree/I rather agree	1081 (58.8)	483 (56.7)	0.299	1.11 [0.94–1.30]

## Data Availability

Aggregated data can be found in the supplement. Non-aggregated data may not be provided for data protections reasons to rule out identification of any participant.

## Supplementary Materials

The following supporting information can be downloaded at: https://www.mdpi.com/article/10.3390/healthcare11111602/s1, Figure S1: Sources of Information regarded as particularly trustworthy for information on medical treatment of COVID-19, relative answer frequencies; Figure S2: Information Barriers: relative answer frequencies for physicians and non-physicians; Figure S3: Media usage to gather information on COVID-19 vaccination, non-physicians vs. physicians, relative frequencies; Table S1: Full questionnaires (Study 1) [21,22,24,25]; Table S2: Appliance of the CHERRIES checklist, table adapted from the Cherries checklist [39]; Table S3: Results of informational Needs and Strategies survey (Study 1).
