# Supplementary material for: Locating Medical Information during an Infodemic: Information Seeking Behavior and Strategies of Health-Care Workers in Germany
Source: Healthcare (Basel). 2023 May 30;11(11):1602. doi: 10.3390/healthcare11111602 (PMC10252231; doi:10.3390/healthcare11111602)
Supplement: Supplementary file 1 [file healthcare-11-01602-s001.zip › healthcare-2382777-supplementary.pdf]

Supplemental Table S1. Full questionnaires (study 1). COVID-19 Informational needs and Strategies Surveys developed within the CEOsys network, the questionnaires were also used in partially modified form in our other studies on information behavior of HCWs [1, 2]. COVID-19 Vaccination Acceptance and Hesitance Study: Questionnaire originally published in 2021 and 2022.[3, 4].

| **COVID-19 INFORMATIONAL NEEDS AND STRATEGIES SURVEY**<br><br>**HCW in hospitals and doctor's practices**<br>**<u>Question 1:</u> In which setting do you primarily care for COVID-19 patients?** |
|---|
| (1) Inpatient care / surgery - (2) Emergency medicine / emergency room - (3) Medical practice / medical care center - (4) Home visits / on-call service – If answer 1 or 2 is selected, question 5 is provided |
| **<u>Question 2:</u> Which professional group do you belong to?** |
| (1) Resident - (2) 'Specialist Physician - (3) Nursing staff - (4) Non-physician medical personnel in rescue service - (5) Other non-physician medical personnel (e.g. medical technical employees) – If answer 1 or 2 is selected, question 4 is provided |
| **<u>Question 3:</u> Do you hold an organizational or managerial position?** |
| (1) yes (2) No |
| **<u>Question 4:</u> In which specialty do you work?** |
| (1) Family medicine - (2) Anesthesiology - (3) Occupational medicine - (4) Ophthalmology - (5) Surgery - (6) Gynecology - (7) Skin and venereal diseases - (8) ENT - (9) Human genetics - (10) Hygiene - (11) Internal Medicine - (12) Child and Adolescent Psychiatry - (13) Laboratory Medicine - (14) Microbiology / Virology - (15) Oral and maxillofacial surgery - (16) Neurosurgery - (17) Neurology - (18) nuclear medicine - (19) public health - (20) palliative medicine - (21) pathology (22) pharmacology - (23) physical and rehabilitative medicine - (24) Psychiatry and Psychotherapy (25) Pediatrics - (26) Radiology - (27) Urology - (28) Dentistry - (29) Other -> if selected free text field appears |
| **<u>Question 5:</u> What type of facility do you work at?** |
| (1) Hospital for basic/regular care - (2) Maximum care hospital - (3) Hospital for specialized care<br>(4) Specialized hospital - (5) Other -> if selected, free text field appears |
| **<u>Question 6:</u> Please prioritize in which pandemic-related topics you would particularly like information.** |
| Specific population groups<br>(1) General population - (2) Health care professionals (physicians, nurses, other health care professionals) - (3) Teachers/educators - (4) children - (5) vulnerable group - (6) Other-> if selected, free text field appears<br>(7) Definition of vulnerable group - (8) Prevention (hygiene, spacing, mobility, vaccination, etc.)<br>(9) Control (testing, follow-up) - (10) Diagnostics - (11) Avoidance of side effects of drug treatment<br>(12) Long-term effects of COVID-19 - (13) Mental health during COVID-19<br>Dealing with different places<br>(14) Schools / day care centers - (15) health care facilities (nursing homes, hospitals) -<br>(16) public places (supermarkets, markets, authorities) - (17) recreation / leisure (parks, sports, restaurants, theaters, other recreational facilities) |
| **<u>Question 7:</u> What other issues regarding COVID-19 in critical care, in addition to the topics mentioned above, are particularly relevant with regard to your daily work and should be addressed in a comprehensive knowledge and evidence ecosystem?** |
| Free text |
| **<u>Question 8:</u> Which of the following platforms and channels do you or would you use to become aware of newly generated evidence/treatment recommendations?** |
| Online Community or Social Media<br>(1) Twitter - (2) Facebook - (3) Instagram - (4) Youtube - (5) Messenger channel (e.g. Telegram)<br>(6) Networking in a research portal (e.g., ResearchGate) - (7) Other (online/social media) if selected free text field appears<br>Campaigns or advertisements via<br>(8) automated newsfeed (RSS feed) - (9) Email/newsletter - (10) Professional journals - (11) Television - (12) Radio - (13) Daily/weekly press - (14) Internet pages of the Robert Koch Institute, AWMF, Federal Ministry of Health, etc. - (15) Professional societies/medical associations<br>(16) Other (campaigns/advertisements), if selected free text field appears |

**<u>Question 9:</u> Particularly with regard to the dynamically developing evidence and knowledge situation during a pandemic, which strategies are the most effective for you to gain knowledge of specific content and put it into practice?**

Online or print material for active knowledge acquisition (e.g., CEOsys website, special apps, journals).
(1) Freely available long version (original work) - (2) Freely available short version (adapted to target group in terms of content/language) - (3) Overviews with action algorithms - (4) Poster
(5) Provision of a freely usable training lecture - (6) Contributions to professional journals -
(7) Mobile support system for clinical decision-making (e.g., Amboss® app, UpToDate app)
Online material for passive knowledge acquisition (accessible at any time)
(8) Power point presentations with voiceover - (9) Podcast in the form of interviews with experts or verbal presentation of content - (10) Videos in the form of reports and audiovisual presentation of contents
Continuing education
(11) Congresses (face-to-face events) - (12) Subject-specific continuing education events (face-to-face event) - (13) Continuing education events directly at the workplace (face-to-face event) -
(14) Webinar (online format with interaction options, takes place at a defined time) -
(15) Digital Journal club (discussion with experts from CEOsys with the possibility to ask questions)
Documentation and quality management - (16) Reminders and quality markers integrated in (digital) documentation system - (17) Checklists (e.g. at the patient's bedside) - (18) Audit and feedback of daily practice by in-house expertise - (19) audit and feedback of daily practice by external expertise - (20) individual reward system - (21) Publish quality indicators to allow comparison with other facilities - (22) Other, if selected free text field appears
Only if an option of 1, 2,3,4,6 or 22 is selected, question 10 appears

**<u>Question 10:</u> Regardless of the exact text format, what type of reading material/informational text distribution is most likely to appeal to you?**

(1) Articles made available in digital form only on a website/app for direct reading. - (2) Articles made available on a website as PDFs with the option of printing. - (3) Analog print material made available.

**<u>Question 11:</u> If you had to choose a format for brief summaries of evidence syntheses, what would it be?**

Summary with interpretation
(1) Tabular - (2) Continuous text - (3) Key points - (4) Graphical/Infographic
Summary without interpretation
(5) Tabular -(6) continuous text - (7) bullet points - (8) Graphic/Infographic

**<u>Question 12:</u> Which organizations (source of information) do you rate as most trustworthy regarding information on COVID-19 medical therapy?**

(1) Cochrane Collaboration - (2) German Network for Evidence-Based Medicine (EbM Network) -
(3) Professional societies (e.g. DIVI, DGAI, DGIIN) - (4) Program for National Health Care Guidelines (cooperation of German Medical Association, KBV, AWMF) - (5) World Health Organization (WHO)
(6) Robert Koch Institute - (7) Federal Center for Health Education - (8) Other, if selected free text field appears

**<u>Question 13:</u> What is your preferred information dissemination strategy that the CEOsys Consortium will use to disseminate newly generated evidence syntheses and guidelines?**

(1) Topics and content are actively pushed to you as the target group; e.g., via email or social media message with a link to the full text/summaries or also via your superiors or organizational management of the institution without you having to become active for this ("push strategy").
(2) Topics and content are freely available. The duration, frequency and level of detail of the information provided is up to you. The information is provided on the basis of topics previously defined by your feedback ("pull strategy").

**<u>Question 14:</u> Will you use a feedback option, i.e., a way to provide feedback to the CEOsys Consortium or other organizations that produce evidence syntheses/guidelines (e.g., AWMF-coordinated nationwide guideline project groups)?**

(1) yes, I will - (2) rather yes - (3) rather no - (4) no, I will not

**<u>Question 15:</u> What barriers do you face to acquiring evidence-based knowledge?**

(1) I have too little time in my daily work to deal with evidence syntheses. - (2) The independent acquisition of evidence-based knowledge is not encouraged by my superiors. - (3) I have no experience in dealing with evidence syntheses. - (4) I am unsure/don't know where or how to access reliable evidence syntheses. - (5) Access to evidence syntheses is too cumbersome (e.g., paid subscriptions/memberships). - (6) The language in evidence syntheses is too complex and difficult for me to understand. - (7) The content in evidence syntheses is not well matched to my

target audience (e.g., in terms of prior knowledge, relevance). - (8) I reject the acquisition of knowledge from evidence syntheses, because in my opinion the contents are not practicable in everyday life (e.g. too undifferentiated, not applicable to the individual). - (9) Other, if selected free text field appears

**HCW IN PUBLIC HEALTH ORGANIZATIONS**

**Question 1: In what role are you primarily responsible for handling COVID-19?**

(1) Politician - (2) Research / Science - (3) Physician, practically active - (4) Physician, administratively active - (5) Non-physician health care professionals, hands-on - (6) Non-physician health care professional, administrative - (7) Other function in administration/organization - (8) Other -> if selected, free text field appears

**Question 2: In what setting are you professionally involved with COVID-19?**

(1) Federal authority - (2) State authority - (3) Local health authority - (4) Health policy (party work, parliamentary work) - (5) Association (KV, KBV, BVÖGD etc.) - (6) Professional society - (7) Hospital organization / management - (8) Other, if selected free text field appears

**Question 3: Do you hold a managerial position?**

(1) yes (2) No

**Question 4: Please prioritize in which pandemic-related topics you would particularly like information.**

Specific population groups
(1) General population - (2) Health care professionals (physicians, nurses, other health care professionals) - (3) Teachers/educators - (4) children - (5) vulnerable group - (6) Other-> if selected, free text field appears
(7) Definition of vulnerable group - (8) Prevention (hygiene, spacing, mobility, vaccination, etc.)
(9) Control (testing, follow-up) - (10) Diagnostics - (11) Avoidance of side effects of drug treatment
(12) Long-term effects of COVID-19 - (13) Mental health during COVID-19
Dealing with different places
(14) Schools / day care centers - (15) health care facilities (nursing homes, hospitals) -
(16) public places (supermarkets, markets, authorities) - (17) recreation / leisure (parks, sports, restaurants, theaters, other recreational facilities)

**Question 5: In terms of your day-to-day work, what other issues regarding COVID-19, in addition to the topic areas mentioned above, are particularly relevant and should be addressed in a comprehensive knowledge and evidence ecosystem?**

Free text

**Question 6: Please rate the following statements about travel restrictions during the pandemic according to your current knowledge.**

There should…
(1) ...be all border crossings be prohibited. - (2) ...partial travel restrictions be imposed (e.g. for travelers from risk areas, air travelers). - (3) ...travelers be controlled by screening for COVID-19 specific symptoms (symptomless → entry possible, symptoms → entry not possible). (4) ...travelers be tested for COVID-19 infection by PCR test.(negative test → entry possible, positive test→ entry not possible). (5) ...travelers be required to undergo 14 days of observation in quarantine before they are allowed to move freely in the country of entry, even if the PCR test is negative.

**Question 7: Which of the following platforms and channels do you or would you use to become aware of newly generated evidence/treatment recommendations?**

Online Community or Social Media
(1) Twitter - (2) Facebook - (3) Instagram - (4) Youtube - (5) Messenger channel (e.g. Telegram)
(6) Networking in a research portal (e.g., ResearchGate) - (7) Other (online/social media) if selected free text field appears
Campaigns or advertisements via
(8) automated newsfeed (RSS feed) - (9) Email/newsletter - (10) Professional journals - (11) Television - (12) Radio - (13) Daily/weekly press - (14) Internet pages of the Robert Koch Institute, AWMF, Federal Ministry of Health, etc. - (15) Professional societies/medical associations
(16) Other (campaigns/advertisements), if selected free text field appears

**Question 8: Particularly with regard to the dynamically developing evidence and knowledge situation during a pandemic, which strategies are the most effective for you to gain knowledge of specific content and put it into practice?**

Online or print material for active knowledge acquisition (e.g., CEOsys website, special apps, journals).
(1) Freely available long version (original work) - (2) Freely available short version (adapted to target group in terms of content/language) - (3) Overviews with action algorithms - (4) Poster
(5) Provision of a freely usable training lecture - (6) Contributions to professional journals -
(7) Mobile support system for clinical decision-making (e.g., Amboss® app, UpToDate app)

Online material for passive knowledge acquisition (accessible at any time)
(8) Power point presentations with voiceover - (9) Podcast in the form of interviews with experts or verbal presentation of content - (10) Videos in the form of reports and audiovisual presentation of contents
Continuing education
(11) Congresses (face-to-face events) - (12) Subject-specific continuing education events (face-to-face event) - (13) Continuing education events directly at the workplace (face-to-face event) -
(14) Webinar (online format with interaction options, takes place at a defined time) -
(15) Digital Journal club (discussion with experts from CEOsys with the possibility to ask questions)
Documentation and quality management - (16) Reminders and quality markers integrated in (digital) documentation system - (17) Checklists (e.g. at the patient's bedside) - (18) Audit and feedback of daily practice by in-house expertise - (19) audit and feedback of daily practice by external expertise - (20) individual reward system - (21) Publish quality indicators to allow comparison with other facilities - (22) Other, if selected free text field appears
Only if an option of 1, 2,3,4,6 is selected, question 9 appears

**<u>Question 9:</u> Regardless of the exact text format, what type of reading material/informational text distribution is most likely to appeal to you?**

(1) Articles made available in digital form only on a website/app for direct reading. - (2) Articles made available on a website as PDFs with the option of printing. - (3) Analog print material made available.

**<u>Question 10:</u> If you had to choose a format for brief summaries of evidence syntheses, what would it be?**

Summary with interpretation
(1) Tabular - (2) Continuous text - (3) Key points - (4) Graphical/Infographic
Summary without interpretation
(5) Tabular -(6) continuous text - (7) bullet points - (8) Graphic/Infographic

**<u>Question 11:</u> Which organizations (source of information) do you rate as most trustworthy regarding information on COVID-19 medical therapy?**

(1) Cochrane Collaboration - (2) German Network for Evidence-Based Medicine (EbM Network) -
(3) Professional societies (e.g. DIVI, DGAI, DGIIN) - (4) Program for National Health Care Guidelines (cooperation of German Medical Association, KBV, AWMF) - (5) World Health Organization (WHO)
(6) Robert Koch Institute - (7) Federal Center for Health Education - (8) Other, if selected free text field appears

**<u>Question 12:</u> How do you determine whether information is trustworthy?**

(1) Information sources are indicated. - (2) The information is displayed with high relevance in a Google search. - (3) Authors are named. - (4) The information is up-to-date.
(5) Funding and possible interests are disclosed. - (6) The information has been produced by an expert in the subject area. - (7) The procedure for preparing the information is described (e.g. research of current scientific studies). - (8) The information is attractively designed and the design appears serious. (9) Other, if selected free text field appears

**<u>Question 13:</u> What is your preferred information dissemination strategy that the CEOsys Consortium will use to disseminate newly generated evidence syntheses and guidelines?**

(1) Topics and content are actively pushed to you as the target group; e.g., via email or social media message with a link to the full text/summaries or also via your superiors or organizational management of the institution without you having to become active for this ("push strategy").
(2) Topics and content are freely available. The duration, frequency and level of detail of the information provided is up to you. The information is provided on the basis of topics previously defined by your feedback ("pull strategy").

**<u>Question 14:</u> Will you use a feedback option, i.e., a way to provide feedback to the CEOsys Consortium or other organizations that produce evidence syntheses/guidelines (e.g., AWMF-coordinated nationwide guideline project groups)?**

(1) yes, I will - (2) rather yes - (3) rather no - (4) no, I will not

**<u>Question 15:</u> What barriers do you face to acquiring evidence-based knowledge?**

(1) I have too little time in my daily work to deal with evidence syntheses. - (2) The independent acquisition of evidence-based knowledge is not encouraged by my superiors. - (3) I have no experience in dealing with evidence syntheses. - (4) I am unsure/don't know where or how to access reliable evidence syntheses. - (5) Access to evidence syntheses is too cumbersome (e.g., paid subscriptions/memberships). - (6) The language in evidence syntheses is too complex and difficult for me to understand. - (7) The content in evidence syntheses is not well matched to my target audience (e.g., in terms of prior knowledge, relevance). - (8) I reject the acquisition of knowledge from evidence syntheses, because in my opinion the contents are not practicable in

everyday life (e.g. too undifferentiated, not applicable to the individual). - (9) Other, if selected free text field appears

**COVID-19 VACCINATION ACCEPTANCE AND HESITANCY STUDY**

**Section 1: Basic data / demographics**

**Sex**

female / male / no answer

**Age**

≤20 years / 21-30 years / 31 - 40 years / 41 - 50 years / 51 - 60 years / ≥61 years / no answer

**Profession**

Certified nurse / Non-examined nurse / Medical specialist (Medical technical assistants, surgical assistant etc.) / Resident physician / specialized physician / Consultant physician / Chief physician / administrative staff / Non-physician staff in the rescue service / Trainee / student of human medicine / Student of dentistry / other – Free text / no answer

**Work Setting**

Maximum-care hospital; university hospital / Hospitals of other care levels / Medical practice; Medical care center / Rescue service / nursing home; retirement home / Outpatient nursing service / other – free text / no answer

**You indicated that you work in an inpatient setting. Do you work in an intensive care unit in this setting? If work setting 1 or 2 have been selected.**

yes / no

**I work with COVID-19 positive patients**

Never / under 50% of my working days / over 50% of my working days (but not every working day) / on each of my working days / no answer

**I work in the following federal district:**

Bavaria / Baden-Wuerttemberg / Berlin / Brandenburg / Bremen / Hamburg / Hesse / Mecklenburg-Western Pomerania / Lower Saxony / North Rhine-Westphalia / Rhineland-Palatinate / Saarland / Saxony / Saxony-Anhalt / Schleswig-Holstein / Thuringia / no answer

**Section 2: Vaccinations in general**

**Please rate the following statements about vaccinations in general:**

**I make sure to keep my vaccinations up to date.**

I do not agree at all / I rather do not agree / I neither agree nor disagree / I rather agree / I fully agree / no answer

**I receive flu (influenza) vaccinations regularly.**

I do not agree at all / I rather do not agree / I neither agree nor disagree / I rather agree / I fully agree / no answer

**I feel well informed about vaccines in general.**

I do not agree at all / I rather do not agree / I neither agree nor disagree / I rather agree / I fully agree / no answer

**I am generally afraid of adverse effects of vaccinations.**

I do not agree at all / I rather do not agree / I neither agree nor disagree / I rather agree / I fully agree / no answer

**I think vaccinations are generally effective.**

I do not agree at all / I rather do not agree / I neither agree nor disagree / I rather agree / I fully agree / no answer

**I trust vaccines in general.**

I do not agree at all / I rather do not agree / I neither agree nor disagree / I rather agree / I fully agree / no answer

**I believe that the pharmaceutical industry puts profit motives over the safety of vaccines.**

I do not agree at all / I rather do not agree / I neither agree nor disagree / I rather agree / I fully agree / no answer

**I trust the regulatory authorities of vaccines in Germany in general.**

I do not agree at all / I rather do not agree / I neither agree nor disagree / I rather agree / I fully agree / no answer

**I already had a serious vaccine adverse effect that required medical treatment.**

yes / no / no answer, if yes -> free text

**Section 3 Vaccinations against COVID-19**

**I have already received one or two vaccinations against COVID-19 or already have a vaccination appointment.**

yes / no / no answer

**In my personal environment (family, friends, colleagues) there are people who have already received one (or two) vaccinations.**

yes / no / I do not know / no answer

**I want to get vaccinated against COVID-19. (Questions opens only if participant indicates not to have been vaccinated yet / if participant clicked "no answer".)**

yes / no / undecided / no answer

**I do not want to get vaccinated until I see that a lot of people who have been vaccinated have tolerated it. (Question only opens, if "undecided" or "no answer" have been indicated.**

I do not agree at all / I rather do not agree / I neither agree nor disagree / I rather agree / I fully agree / no answer

**In my personal environment (family, friends, colleagues) there were people suffering from COVID-19.**

yes / no / I do not know / no answer

**You have indicated that there were COVID-19 patients in your personal environment. Please answer the following questions: (Question only opens, if "yes" has been indicated.)**
**One / more persons of the above have been hospitalized in connection with COVID-19 disease**

yes / no / I do not know / no answer

**One / more persons of the above have been in an intensive care unit in connection with the COVID-19 disease**

yes / no / I do not know / no answer

**One / more persons of the above have died in connection with COVID-19 disease**

yes / no / I do not know / no answer

**In my personal environment (family, friends, colleagues) there are people for whom a COVID-19 disease would probably be severe.**

yes / no / I do not know / no answer

**Please answer the following questions on COVID-19 / vaccinations against COVID-19:**

**In case of COVID-19 disease, I fear a severe course of the disease for myself**

I do not agree at all / I rather do not agree / I neither agree nor disagree / I rather agree / I fully agree / no answer

**I am afraid of getting infected with COVID-19 in my professional environment.**

I do not agree at all / I rather do not agree / I neither agree nor disagree / I rather agree / I fully agree / no answer

**I am afraid of becoming infected with COVID-19 in a private environment.**

I do not agree at all / I rather do not agree / I neither agree nor disagree / I rather agree / I fully agree / no answer

**I feel well informed about COVID-19 vaccines.**

I do not agree at all / I rather do not agree / I neither agree nor disagree / I rather agree / I fully agree / no answer

**I am afraid of short-term adverse effects of COVID-19 vaccines.**

I do not agree at all / I rather do not agree / I neither agree nor disagree / I rather agree / I fully agree / no answer

**I am afraid of long-term adverse effects from COVID-19 vaccines.**

I do not agree at all / I rather do not agree / I neither agree nor disagree / I rather agree / I fully agree / no answer

**I believe the COVID-19 vaccines are effective.**

I do not agree at all / I rather do not agree / I neither agree nor disagree / I rather agree / I fully agree / no answer

**I am concerned about what I consider novel mechanisms of action in COVID-19 vaccines.**

I do not agree at all / I rather do not agree / I neither agree nor disagree / I rather agree /

I fully agree / no answer

**The development and approval of COVID-19 vaccines are moving too fast for me.**

I do not agree at all / I rather do not agree / I neither agree nor disagree / I rather agree / I fully agree / no answer

**I generally trust the COVID-19 vaccines.**

I do not agree at all / I rather do not agree / I neither agree nor disagree / I rather agree / I fully agree / no answer

**I believe the pharmaceutical industry cares more about its profit motives than safety of COVID-19 vaccines.**

I do not agree at all / I rather do not agree / I neither agree nor disagree / I rather agree / I fully agree / no answer

**I trust the European regulatory authorities of COVID-19 vaccines used in Germany.**

I do not agree at all / I rather do not agree / I neither agree nor disagree / I rather agree / I fully agree / no answer

**I generally trust in the German health care politics.**

I do not agree at all / I rather do not agree / I neither agree nor disagree / I rather agree / I fully agree / no answer

**I generally trust in the medical opinion of physicians.**

I do not agree at all / I rather do not agree / I neither agree nor disagree / I rather agree / I fully agree / no answer

**I favor an introduction of compulsory vaccination for health care professionals.**

I do not agree at all / I rather do not agree / I neither agree nor disagree / I rather agree / I fully agree / no answer

**In case of approval by the European and German authorities, I would also be vaccinated with a vaccine not developed in America or Europe.**

I do not agree at all / I rather do not agree / I neither agree nor disagree / I rather agree / I fully agree / no answer

**If you are concerned about short-term adverse effects, what are they?**

Free text

**If you are concerned about long-term adverse effects, what are they?**

Free text

**Knowledge**

**Which of the statements about COVID-19 vaccines do you think are correct?**

**m-RNA vaccines stimulate the formation of protein structures that mimic parts of the viral envelope.**

true / false / I do not know / no answer

**m-RNA vaccines alter the genetic material**

true / false / I do not know / no answer

**COVID-19 vaccination can cause me to shed infectious viruses**

true / false / I do not know / no answer

**m-RNA is rapidly degraded in the body**

true / false / I do not know / no answer

**My primary care physician has given me ...**

advise to get vaccinated against COVID-19 / advise not to get vaccinated COVID-19 / We have not discussed the vaccination so far. / We have addressed the vaccination; my primary care physician has not made a recommendation for or against the vaccination. / no answer

**The majority of my colleagues have / would like to**

get vaccinated against COVID-19 / not get vaccinated against COVID-19 / not yet decided whether to get vaccinated / we have not discussed vaccination so far / answer

**The majority of my family / friends have or would like to**

get vaccinated against COVID-19 / not get vaccinated against COVID-19 / not yet decided whether to get vaccinated / we have not discussed vaccination so far / no answer

| I have given the majority of my patients (without contraindication) ... |
|---|
| advise to get vaccinated against COVID-19 / advise not to get vaccinated COVID-19 / We have not discussed the vaccination so far / We have addressed the vaccination; I have not made a recommendation for or against the vaccination / no answer |
| **I have given my family / friends** |
| advise to get vaccinated against COVID-19 / advise not to get vaccinated COVID-19 / We have not discussed the vaccination so far / We have addressed the vaccination; I have not made a recommendation for or against the vaccination / no answer |
| **Because of the COVID-19 pandemic, I, or my family, have suffered economic losses.** |
| yes / no / I do not know / no answer |
| **From what media do you get your information on COVID-19 vaccination?** |
| Daily newspapers, weekly magazines (print) / Daily newspapers, weekly magazines (online) / Scientific journals (print) / Scientific journals (online) / Television / radio / social networks (e.g., Facebook) / messenger services (e.g., Twitter, Telegram) / Online video platforms (e.g., YouTube) / Websites / information portals of government health authorities (e.g., RKI, WHO) / Other -> Free text |

Supplemental Table S2. Appliance of the CHERRIES checklist, table adapted from the Cherries checklist [5].

| **Item** Category | **Reported in manuscript** |
|---|---|
| **Design** | |
| Describe survey design | √ |
| **IRB (Institutional Review Board) approval and informed consent process** | |
| IRB approval | √ |
| Informed consent | √ |
| Data protection | √ |
| **Development and pre-testing** | |
| Development and testing | √ |
| **Recruitment process and description of the sample having access to the questionnaire** | |
| Open survey versus closed survey | √ |
| Contact mode | √ |
| Advertising the survey | √ |
| **Survey administration** | |
| Web/E-mail | √ |
| Context | √ |
| Mandatory/voluntary | √ |
| Incentives | √ |
| Time/Date | √ |
| Randomization of items or questionnaires | √ |
| Adaptive questioning | √ |

| **Item** Category | **Reported in manuscript** |
|---|---|
| Number of Items | √ |
| Number of screens (pages) | √ |
| Completeness check | √ |
| Review step | √ |
| **Response rates** | |
| Unique site visitor | n/a |
| View rate (Ratio of unique survey visitors/unique site visitors) | n/a |
| Participation rate (Ratio of unique visitors who agreed to participate/unique first | n/a |
| Completion rate (Ratio of users who finished the survey/users who agreed to | √ |
| **Preventing multiple entries from the same individual** | |
| Cookies used | √ |
| IP check | √ |
| Log file analysis | √ |
| Registration | √ |
| **Analysis** | |
| Handling of incomplete questionnaires | √ |
| Questionnaires submitted with an atypical timestamp | √ |
| Statistical correction | √ |

Supplemental Table S3. Results of informational Needs and Strategies survey (study 1), grouped by physicians / non-physicians and patient care / non-patient care, Reference for Odds ratios: non-physicians.

| **Informational Needs and Strategies Survey, N=413** | | | | | | | | |
|---|---|---|---|---|---|---|---|---|
| **Item** | **Physicians, N=299; N (%)** | **Non-Physicians, N=114; N (%)** | **p** | **Odds ratio [95% CI]** | **Patient care, N=303; N (%)** | **Non-patient care, N=110; N (%)** | **p** | **Odds ratio [95% CI]** |
| **Which of the following platforms and channels do you or would you use to become aware of newly generated evidence/treatment recommendations** | | | | | | | | |
| **Online Community or Social Media** | | | | | | | | |
| *Missing* | *0* | *0* | | | *0* | *0* | | |
| Twitter | 34 (11.4) | 11 (9.6) | 0.616 | 1.20 [0.59 – 2.46] | 34 (11.2) | 11 (10.0) | 0.725 | 1.14 [0.55 – 2.33] |
| Facebook | 42 (14.0) | 33 (28.9) | <0.001 | 0.40 [0.24 – 0.67] | 57 (18.8) | 18 (16.4) | 0.568 | 1.18 [0.66 – 2.12] |
| Instagram | 15 (5.0) | 18 (15.8) | <0.001 | 0.28 [0.14 – 0.58] | 26 (8.6) | 7 (6.4) | 0.463 | 1.38 [0.58 – 3.28] |
| Youtube | 52 (17.4) | 30 (26.3) | 0.042 | 0.59 [0.35 – 0.98] | 62 (20.5) | 20 (18.2) | 0.608 | 1.16 [0.66 – 2.03] |
| Messenger channel (e.g. Telegram) | 49 (16.4) | 12 (10.5) | 0.133 | 1.67 [0.85 – 3.26] | 47 (15.5) | 14 (12.7) | 0.481 | 1.26 [0.66 – 2.03] |
| Networking in a research portal (e.g., ResearchGate) | 127 (42.5) | 26 (22.8) | <0.001 | 2.50 [1.52 – 4.10] | 101 (33.3) | 52 (47.3) | 0.010 | 0.56 [0.36 – 0.87] |
| Other | 48 (16.1) | 15 (13.2) | 0.464 | 1.26 [0.68 – 2.36] | 48 (15.8) | 15 (13.6) | 0.582 | 1.19 [0.64 – 2.23] |
| **Campaigns or advertisements via** | | | | | | | | |
| automated newsfeed (RSS feed) | 30 (10.0) | 12 (10.5) | 0.882 | 0.95 [0.47 – 1.92] | 28 (9.2) | 14 (12.7) | 0.300 | 0.70 [0.35 – 1.38] |
| Email/newsletter | 168 (56.2) | 53 (46.5) | 0.077 | 1.48 [0.96 – 2.28] | 171 (56.4) | 50 (45.5) | 0.048 | 1.55 [1.00 – 2.41] |
| Professional journals | 165 (55.2) | 23 (20.2) | <0.001 | 4.87 [2.92 – 8.12] | 148 (48.8) | 40 (36.4) | 0.024 | 1.67 [1.07 – 2.62] |
| Television | 69 (23.1) | 65 (57.0) | <0.001 | 0.23 [0.14 – 0.36] | 91 (30.0) | 43 (49.1) | 0.082 | 0.67 [0.42 – 1.05] |
| Radio | 58 (19.4) | 46 (40.4) | <0.001 | 0.36 [0.22 – 0.57] | 72 (23.8) | 32 (29.1) | 0.270 | 0.76 [0.47 – 1.24] |
| Daily/weekly press | 65 (21.7) | 37 (32.5) | 0.024 | 0.58 [0.36 – 0.93] | 74 (24.4) | 28 (25.5) | 0.830 | 0.95 [0.57 – 1.56] |
| Internet pages of the Robert Koch Institute, AWMF, Federal Ministry of Health, etc. | 188 (62.9) | 65 (57.0) | 0.275 | 1.28 [0.82 – 1.98] | 173 (57.1) | 80 (72.2) | 0.004 | 0.50 [0.31 – 0.80] |
| Professional societies/medical associations | 149 (49.8) | 15 (13.2) | <0.001 | 6.56 [3.64 – 11.81] | 117 (38.6) | 47 (42.7) | 0.450 | 0.84 [0.54 – 1.31] |
| Other | 3 (1.0) | 3 (2.6) | 0.216 | 0.38 [0.07 – 1.89] | 3 (1.0) | 3 (2.7) | 0.192 | 0.36 [0.07 – 1.79] |
| **Which organizations (source of information) do you rate as most trustworthy regarding information on COVID-19 medical therapy?** | | | | | | | | |
| *Missing* | *19* | *11* | | | *23* | *7* | | |
| Cochrane Collaboration | 78 (27.9) | 5 (4.9) | <0.001 | 7.57 [2.97 – 19.29] | 61 (21.8) | 22 (21.4) | 0.928 | 1.03 [0.59 – 1.78] |
| German Network for Evidence-Based Medicine (EbM Network) | 41 (14.6) | 6 (5.8) | 0.020 | 2.77 [1.14 – 6.74] | 32 (11.4) | 15 (14.6) | 0.407 | 0.76 [0.39 – 1.46] |

| | | | | | | | | |
|---|---|---|---|---|---|---|---|---|
| Professional societies (e.g. DIVI, DGAI, DGIIN) | 139 (49.6) | 16 (15.5) | <0.001 | 5.36 [2.99 – 9.60] | 124 (44.3) | 31 (30.1) | 0.012 | 1.85 [1.14 – 2.99] |
| Program for National Health Care Guidelines (cooperation of German Medical Association, KBV, AWMF) | 92 (32.9) | 11 (10.7) | <0.001 | 4.09 [2.09 – 8.02] | 76 (27.1) | 27 (26.2) | 0.856 | 1.05 [0.63 – 1.75] |
| World Health Organization (WHO) | 69 (24.6) | 42 (40.8) | 0.002 | 0.47 [0.29 – 0.77] | 89 (31.8) | 22 (21.4) | 0.046 | 1.72 [1.01 – 2.93] |
| Robert Koch Institute | 223 (79.6) | 95 (92.2) | 0.004 | 0.33 [0.15 – 0.72] | 229 (81.8) | 89 (86.4) | 0.285 | 0.71 [0.37 – 1.34] |
| Federal Center for Health Education | 24 (8.6) | 28 (27.2) | <0.001 | 0.25 [0.14 – 0.46] | 29 (10.4) | 23 (22.3) | 0.002 | 0.40 [0.22 – 0.73] |
| Other | 9 (3.2) | 6 (5.8) | 0.243 | 0.54 [0.19 – 1.55] | 9 (3.2) | 6 (5.8) | 0.243 | 0.54 [0.19 – 1.55] |
| **What barriers do you face to acquiring evidence-based knowledge** | | | | | | | | |
| *Missing* | *23* | *14* | | | *27* | *10* | | |
| I have too little time in my daily work to deal with evidence syntheses. | 218 (79.0) | 55 (55.0) | <0.001 | 3.08 [1.89 – 5.01] | 200 (72.5) | 73 (73.0) | 0.918 | 0.97 [0.58 – 1.63] |
| The independent acquisition of evidence-based knowledge is not encouraged by my superiors. | 30 (10.9) | 10 (10.0) | 0.809 | 1.10 [0.52 – 2.34] | 34 (12.3) | 6 (6.0) | 0.079 | 2.20 [0.89 – 5.41] |
| I have no experience in dealing with evidence syntheses. | 28 (10.1) | 30 (30.0) | <0.001 | 0.26 [0.15 – 0.47] | 40 (14.5) | 18 (18.0) | 0.405 | 0.75 [0.41 – 1.39] |
| I am unsure/don't know where or how to access reliable evidence syntheses. | 53 (19.2) | 21 (21.0) | 0.699 | 0.89 [0.51 – 1.58] | 61 (22.0) | 13 (13.0) | 0.050 | 1.90 [0.99 – 3.63] |
| Access to evidence syntheses is too cumbersome (e.g., paid subscriptions/memberships). | 99 (35.9) | 17 (17.0) | <0.001 | 2.73 [1.53 – 4.86] | 81 (29.3) | 35 (35.0) | 0.294 | 0.77 [0.47 – 1.25] |
| The language in evidence syntheses is too complex and difficult for me to understand. | 13 (4.7) | 20 (20.0) | <0.001 | 0.20 [0.09 – 0.42] | 21 (7.6) | 12 (12.0) | 0.184 | 0.60 [0.29 – 1.28] |
| The content in evidence syntheses is not well matched to my target audience (e.g., in terms of prior knowledge, relevance). | 31 (11.2) | 17 (17.0) | 0.139 | 0.62 [0.33 – 1.17] | 33 (12.0) | 15 (15.0) | 0.435 | 0.77 [0.40 – 1.49] |
| I reject the acquisition of knowledge from evidence syntheses, because in my opinion the contents are not practicable in everyday life (e.g. too undifferentiated, not applicable to the individual). | 3 (1.1) | 1 (1.0) | 0.942 | 1.09 [0.11 – 10.58] | 1 (0.4) | 3 (3.0) | 0.028 | 0.12 [0.01 – 1.14] |
| Other | 11 (4.0) | 3 (3.0) | 0.656 | 1.342 [0.37 – 4.91] | 13 (4.7) | 1 (1.0) | 0.093 | 4.89 [0.63 – 37.90] |
| **Particularly with regard to the dynamically developing evidence and knowledge situation during a pandemic, which strategies are the most effective for you to gain knowledge of specific content and put it into practice?** | | | | | | | | |
| *Missing* | *10* | *6* | | | *13* | *3* | | |
| Online or print material for active knowledge acquisition (e.g., CEOsys website, special apps, journals). | | | | | | | | |
| Freely available long version (original work) | 77 (26.6) | 11 (10.2) | <0.001 | 3.20 [1.63 – 6.30] | 61 (21.0) | 27 (25.2) | 0.371 | 0.79 [0.47 – 1.33] |
| Freely available short version (adapted to target group in terms of content/language) | 179 (61.9) | 71 (65.7) | 0.485 | 0.85 [0.53 – 1.35] | 175 (60.3) | 75 (70.1) | 0.074 | 0.65 [0.40 – 1.05] |
| Overviews with action algorithms | 190 (65.7) | 32 (29.6) | <0.001 | 4.56 [2.82 – 7.36] | 157 (54.1) | 65 (60.7) | 0.239 | 0.76 [0.49 – 1.20] |
| Poster | 13 (4.5) | 15 (13.9) | 0.001 | 0.29 [0.13 – 0.64] | 19 (6.6) | 9 (8.4) | 0.521 | 0.76 [0.33 – 1.74] |

| | | | | | | | | |
|---|---|---|---|---|---|---|---|---|
| Provision of a freely usable training lecture | 50 (17.3) | 22 (20.4) | 0.480 | 0.82 [0.47 – 1.43] | 53 (18.3) | 19 (17.8) | 0.905 | 1.04 [0.58 – 1.85] |
| Contributions to professional journals | 102 (35.3) | 17 (15.7) | <0.001 | 2.92 [0.47 – 1.43] | 84 (29.0) | 35 (32.7) | 0.470 | 0.84 [0.52 – 1.35] |
| Mobile support system for clinical decision-making (e.g., Amboss ® app, UpToDate app) | 81 (28.0) | 12 (11.1) | <0.001 | 3.12 [1.62 – 5.98] | 78 (26.9) | 15 (14.0) | 0.007 | 2.26 [1.23 – 4.13] |
| Online material for passive knowledge acquisition (accessible at any time) | | | | | | | | |
| Power point presentations with voiceover | 66 (22.8) | 21 (19.4) | 0.467 | 1.23 [0.71 – 2.13] | 63 (21.7) | 24 (22.4) | 0.880 | 0.96 [0.56 – 1.64] |
| Podcast in the form of interviews with experts or verbal presentation of content | 96 (33.2) | 34 (31.5) | 0.743 | 1.08 [0.67 – 1.74] | 97 (33.4) | 33 (30.8) | 0.623 | 1.13 [0.70 – 1.82] |
| Videos in the form of reports and audiovisual presentation of contents | 77 (26.6) | 49 (45.4) | <0.001 | 0.44 [0.28 – 0.69] | 92 (31.7) | 34 (31.8) | 0.991 | 1.00 [0.62 – 1.61] |
| Continuing education | | | | | | | | |
| Congresses (face-to-face events) | 29 (10.0) | 9 (8.3) | 0.608 | 1.23 [0.56 – 2.68] | 29 (10.0) | 9 (8.4) | 0.633 | 1.21 [0.55 – 2.65] |
| Subject-specific continuing education events (face-to-face event) | 45 (15.6) | 11 (10.2) | 0.170 | 1.63 [0.81 – 3.27] | 40 (13.8) | 16 (15.0) | 0.768 | 0.91 [0.49 – 1.70] |
| Continuing education events directly at the workplace (face-to-face event) | 46 (15.9) | 33 (30.6) | 0.001 | 0.43 [0.26 – 0.72] | 61 (21.0) | 18 (16.8) | 0.351 | 1.32 [0.74 – 2.35] |
| Webinar (online format with interaction options, takes place at a defined time) | 140 (48.4) | 43 (39.8) | 0.125 | 1.42 [0.91 – 2.23] | 121 (41.7) | 62 (57.9) | 0.004 | 0.52 [0.33 – 0.81] |
| Digital Journal club (discussion with experts from CEOsys with the possibility to ask questions) | 31 (10.7) | 4 (3.7) | 0.028 | 3.12 1.08 – 9.07] | 28 (9.7) | 7 (6.5) | 0.332 | 1.53 [0.65 – 3.61] |
| Documentation and quality management | | | | | | | | |

Supplemental Figure S1. Sources of Information regarded as particularly trustworthy for information on medical treatment of COVID-19, relative answer frequencies.

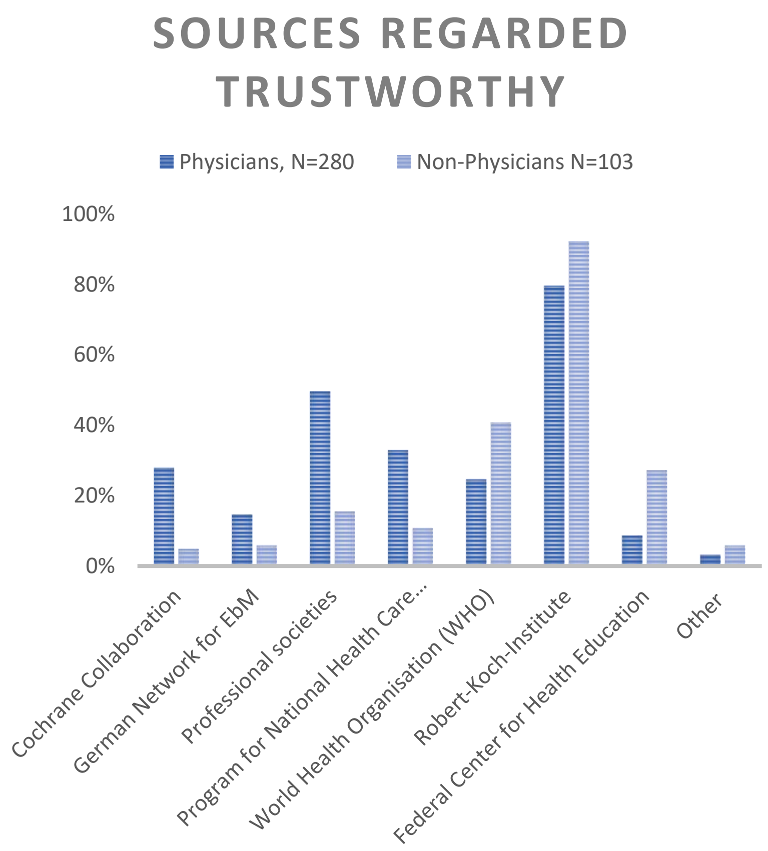

Supplemental Figure S2. Information Barriers: relative answer frequencies for physicians and non-physicians.

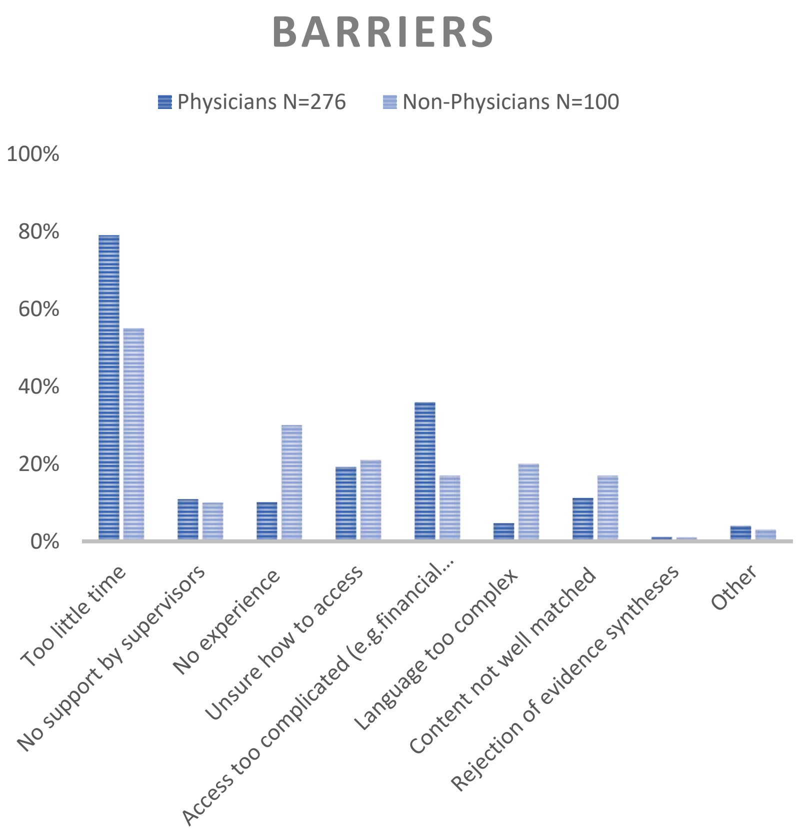

Supplemental Figure S3. Media usage to gather information on COVID-19 vaccination, non-physicians vs. physicians, relative frequencies.

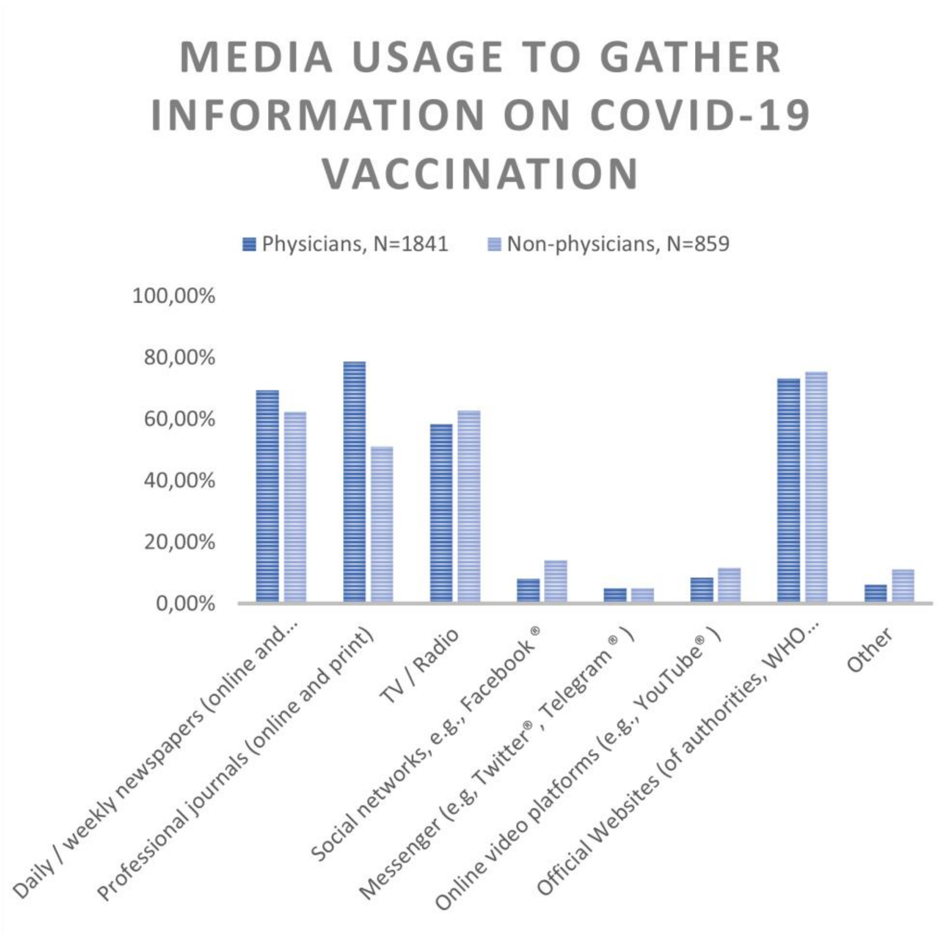


References


1. Seeber, C., et al., *COVID-19-Pandemie: Präferenzen und Barrieren für die Disseminierung von Evidenzsynthesen.* Der Anaesthesist, 2021: p. 1-8.
2. Werner, A., et al., *COVID-19 Intensive Care-Evaluation of Public Information Sources and Current Standards of Care in German Intensive Care Units: A Cross Sectional Online Survey on Intensive Care Staff in Germany.* Healthcare (Basel), 2022. **10**(7).
3. Holzmann-Littig, C., et al., *COVID-19 Vaccination Acceptance and Hesitancy among Healthcare Workers in Germany.* Vaccines, 2021. **9**(7): p. 777.
4. Holzmann-Littig, C., et al., *COVID-19 Vaccines: Fear of Side Effects among German Health Care Workers.* Vaccines (Basel), 2022. **10**(5).
5. Eysenbach, G., *Improving the quality of Web surveys: the Checklist for Reporting Results of Internet E-Surveys (CHERRIES).* J Med Internet Res, 2004. **6**(3): p. e34.
